# Adenosine A_2A_ receptor ligand recognition and signaling is blocked by A_2B_ receptors

**DOI:** 10.18632/oncotarget.24423

**Published:** 2018-02-06

**Authors:** Sonja Hinz, Gemma Navarro, Dasiel Borroto-Escuela, Benjamin F. Seibt, York-Christoph Ammon, Elisabetta de Filippo, Azeem Danish, Svenja K. Lacher, Barbora Červinková, Muhammad Rafehi, Kjell Fuxe, Anke C. Schiedel, Rafael Franco, Christa E. Müller

**Affiliations:** ^1^ PharmaCenter Bonn, Pharmaceutical Institute, Pharmaceutical Chemistry I, University of Bonn, Bonn, Germany; ^2^ Department of Biochemistry and Molecular Biomedicine, Faculty of Biology, University of Barcelona, Barcelona, Spain; ^3^ Department of Neuroscience, Karolinska Institutet, Stockholm, Sweden; ^4^ Centro de Investigación en Red, Enfermedades Neurodegenerativas (CIBERNED), Instituto de Salud Carlos III, Madrid, Spain

**Keywords:** adenosine receptors (ARs), G protein-coupled receptor (GPCR), immuno-oncology, pharmacology, receptor heteromerization

## Abstract

The adenosine receptor (AR) subtypes A_2A_ and A_2B_ are rhodopsin-like G_s_ protein-coupled receptors whose expression is highly regulated under pathological, e.g. hypoxic, ischemic and inflammatory conditions. Both receptors play important roles in inflammatory and neurodegenerative diseases, are blocked by caffeine, and have now become major drug targets in immuno-oncology. By Förster resonance energy transfer (FRET), bioluminescence resonance energy transfer (BRET), bimolecular fluorescence complementation (BiFC) and proximity ligation assays (PLA) we demonstrated A_2A_-A_2B_AR heteromeric complex formation. Moreover we observed a dramatically altered pharmacology of the A_2A_AR when co-expressed with the A_2B_AR (A_2B_ ≥ A_2A_) in recombinant as well as in native cells. In the presence of A_2B_ARs, A_2A_-selective ligands lost high affinity binding to A_2A_ARs and displayed strongly reduced potency in cAMP accumulation and dynamic mass redistribution (DMR) assays. These results have major implications for the use of A_2A_AR ligands as drugs as they will fail to modulate the receptor in an A_2A_-A_2B_ heteromer context. Accordingly, A_2A_-A_2B_AR heteromers represent novel pharmacological targets.

## INTRODUCTION

Adenosine receptors (ARs) are G protein-coupled receptors (GPCRs) activated by the nucleoside adenosine. Four subtypes designated A_1_, A_2A_, A_2B_ and A_3_ARs exist. A_1_ and A_3_ARs preferentially couple to G_i/o_ proteins mediating inhibition of adenylate cyclase (AC) activity, while A_2A_ and A_2B_ receptors couple to G_s/olf_ proteins leading to AC activation and subsequent increase in cAMP formation [1]. In addition, A_2B_ and A_3_ARs were shown to couple to G_q_ proteins which results in phospholipase C activation followed by a rise in inositol trisphosphate levels mediating intracellular calcium release [2–3]. The A_2A_AR is expressed in high density in the caudate-putamen, and at low levels in most other brain regions. In the periphery, the A_2A_AR is highly expressed in cells of the immune system and blood platelets, and at lower levels in many other cells and organs [4]. The A_2B_AR is broadly expressed but mostly at moderate to low levels. A_2A_ and A_2B_ARs are the most closely related AR subtypes with an overall sequence identity of 58% and a similarity of 73% [5]. They are co-expressed on many different cell types and in various organs and tissues, e.g. in heart [6], myeloid cells [7], T-cells [8], blood platelets [9], brown and white adipocytes [10], and in many tumors, e.g. neuroendocrine tumors [11], ovarian cancer [12], and prostate cancer [13]. The expression of A_2A_ and A_2B_ARs and their relative proportion can be markedly altered under pathological conditions [14]. For example, increased A_2A_AR expression is observed in the brains of patients suffering from neurodegenerative diseases [15], in multiple sclerosis and in amyotrophic lateral sclerosis [16–17]. Upon activation of T-lymphocytes the A_2A_AR is considerably upregulated [18]. On the other hand, the expression of A_2B_ARs can be drastically increased in a hypoxia-inducible factor- (HIF1α-) dependent manner under hypoxic conditions, e.g. in inflamed or ischemic tissue, in tumors and cancer cells [19–20]. Hypoxia induction leads to a decrease in A_2A_AR expression while increasing A_2B_AR expression in human umbilical vein endothelial and bronchial smooth muscle cells. Pharmacological responses of A_2A_/A_2B_AR agonists were significantly altered in these cells [21]. The well investigated A_2A_AR subtype, the so-called “high-affinity A_2_AR receptor”, is typically activated by relatively low (nanomolar) concentrations of adenosine, mediating potent anti-inflammatory and immunosuppressant as well as hypotensive and anti-psychotic effects [22]. In contrast, activation of the A_2B_AR subtype, the “low-affinity A_2_AR”, requires high, micromolar adenosine concentrations for activation [4]. Extracellular adenosine levels can rise from basal values of around 100 nM by up to 100-fold reaching concentrations of around 10 µM under pathological, i.e. hypoxic, ischemic or inflammatory conditions [1, 4, 23]. Cell death can lead to the formation of large amounts of extracellular adenosine through enzymatic degradation of released ATP by ectonucleotidases (CD39, CD73), e.g. in solid tumors [24]. Both anti- as well as pro-inflammatory effects have been associated with the A_2B_AR [25], and the reasons for these contradictory results have remained obscure. The physiological significance of the A_2B_AR subtype is scarcely understood so far. During the last decade it has become well accepted that GPCRs are able to form di- or oligomeric assemblies of identical or distinct receptor monomers [26]. Most of these complexes have been detected in transfected living cells using well accepted biophysical techniques such as resonance energy transfer (bioluminescence and Förster resonance energy transfer, BRET and FRET) or bimolecular fluorescence complementation (BiFC) assays [27–28]. Proximity ligation assays (PLA) have been developed for identifying receptor heteromers in native cells and tissues [29]. Heteromer formation may modulate receptor pharmacology such as the affinity and potency of ligands or G protein coupling and signaling [30–31]. Recently, structural models of GPCR oligomers associated with G proteins have been built [32], and the development of heteromer-selective receptor ligands is becoming a promising new research area [33]. The A_2A_AR was reported to form homomeric receptor complexes as well as heteromers with several other GPCRs including dopamine D_2_ and D_3_, cannabinoid CB_1_, nucleotide P2Y_1_ and P2Y_2_, and A_1_ARs [1]. Especially A_2A_-D_2_ heteromeric receptor complexes have been intensively studied since they play a significant role in Parkinson’s disease [34]. However, homo- or heteromer formation of the A_2B_AR subtype has not been demonstrated up to now. Based on the frequent co-expression of the closely related AR subtypes A_2A_ and A_2B_, and considering the up-regulation of the A_2B_AR subtype and the up- or down-regulation of the A_2A_AR under many pathological conditions, the question arises if both receptors could form heteromers and whether this might affect their pharmacology and signaling. Here we demonstrate that A_2A_-A_2B_AR heteromers are formed in living cells by employing FRET, BRET and PLA, and their presence in native tissue was confirmed. Heteromer formation was found to be independent of the presence of agonists or antagonists, and does not require the long C-terminus of the A_2A_AR. Importantly, we demonstrate that A_2A_-A_2B_ heteromerization is the reason for drastically altered pharmacology, in particular for the A_2A_AR, which is completely blocked by the presence of A_2B_AR protein. These results can now help to explain many unexpected or previously misinterpreted observations. They will be of high relevance for recently started drug development programs targeting A_2A_ or A_2B_ARs, in particular in neurodegenerative diseases and immuno-oncology.

## RESULTS

### FRET, BRET and BiFC experiments

**Figure 1:**
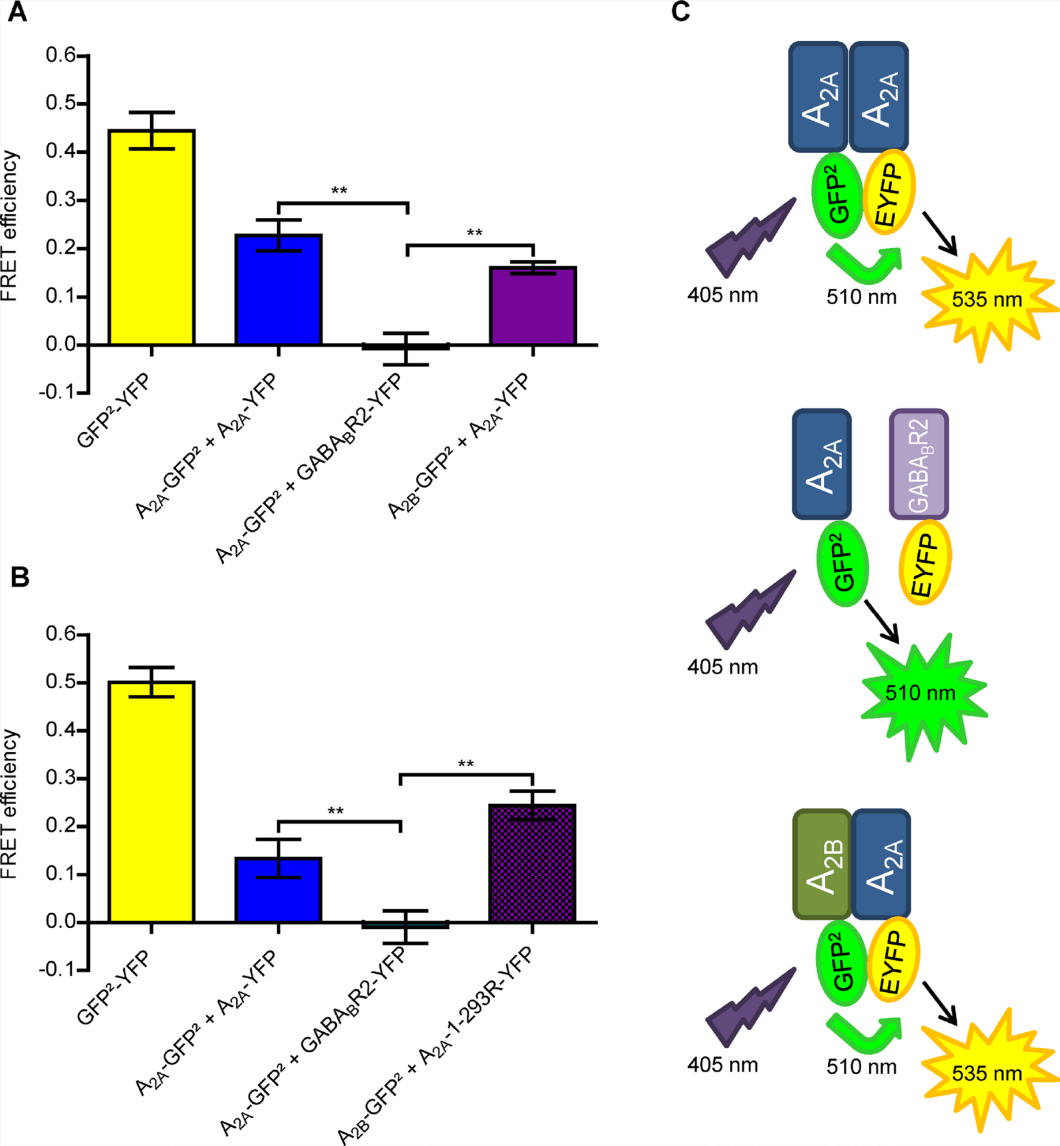
Biophysical assays using A_2A_ and A_2B_ARs fused to FRET donor and acceptor (**A**) FRET efficiencies were calculated by a sensitized emission method in living CHO cells transiently transfected with the different plasmids. Data are means ± SEM of 4–5 independent experiments performed in duplicates. The one-way ANOVA with Dunnett’s post-hoc test showed significant differences in A_2A_-GFP^2^ + A_2A_-EYFP (positive control) or A_2B_-GFP^2^ + A_2A_-EYFP versus the negative control (A_2A_-GFP^2^ + GABA_B_R2-EYFP), ^**^p < 0.01. As an internal control the fusion protein GFP^2^-EYFP was used. (**B**) FRET efficiencies determined in CHO cells transiently transfected with the different plasmids. The same controls were used as shown in (**A**). Data are means ± SEM of 5 independent experiments performed in duplicates. The one-way ANOVA with Dunnett’s post-hoc test showed significant differences between A_2A_-GFP^2^ + A_2A_-EYFP (positive control) or A_2B_-GFP^2^ + A_2A_-1-293-EYFP versus the negative control (A_2A_-GFP^2^ + GABA_B_R2-EYFP), ^**^p < 0.01. (**C**) Schematic representation of the performed FRET experiments with the different donor/acceptor pairs.

FRET is a powerful technique for measuring protein-protein interactions in living cells [35]. To investigate a possible A_2A_-A_2B_AR interaction, FRET experiments were performed in Chinese hamster ovary (CHO-K1) cells transiently transfected with fusion proteins of green fluorescent protein variant 2 (GFP^2^) and enhanced yellow fluorescent protein (EYFP) attached to the C-terminus of the receptors [36] (Figure 1A–1C). The previously described A_2A_-homodimer and the fusion protein GFP^2^-EYFP were employed as positive controls showing FRET efficiencies of 0.23 and 0.44, respectively, similar to those previously reported (A_2A_ homodimer: 0.28, GFP^2^-EYFP: 0.52) (Figure 1A, 1B) [37]. The fusion protein GFP^2^-EYFP displayed a high FRET efficiency due to the very close proximity of donor and acceptor as a result of the short linker between both fluorophores. A clear FRET signal with an efficiency of 0.16 was observed in the co-transfected cells indicating the formation of A_2A_-A_2B_ heteroreceptor complexes (Figure 1A). The pair A_2A_AR and GABA_B2_ receptor [36–37] was employed as a negative control; it showed a very low FRET signal demonstrating the specificity of the observed interactions (Figure 1A–1B). To gain insight into the potential A_2A_-A_2B_ heteromer interface, the C-terminal tail of the A_2A_AR was removed and the resulting construct A_2A_1-293R-EYFP was studied in FRET experiments as an acceptor fluorophore in combination with the A_2B_-GFP^2^ donor fluorophore. The results indicated that the A_2A_AR that was lacking the C-terminal domain was still fully able to form heteromers with the A_2B_AR (FRET efficiency 0.24, Figure 1B) suggesting that different receptor domains, possibly helical domains, have to be involved in heteromer formation.

**Figure 2:**
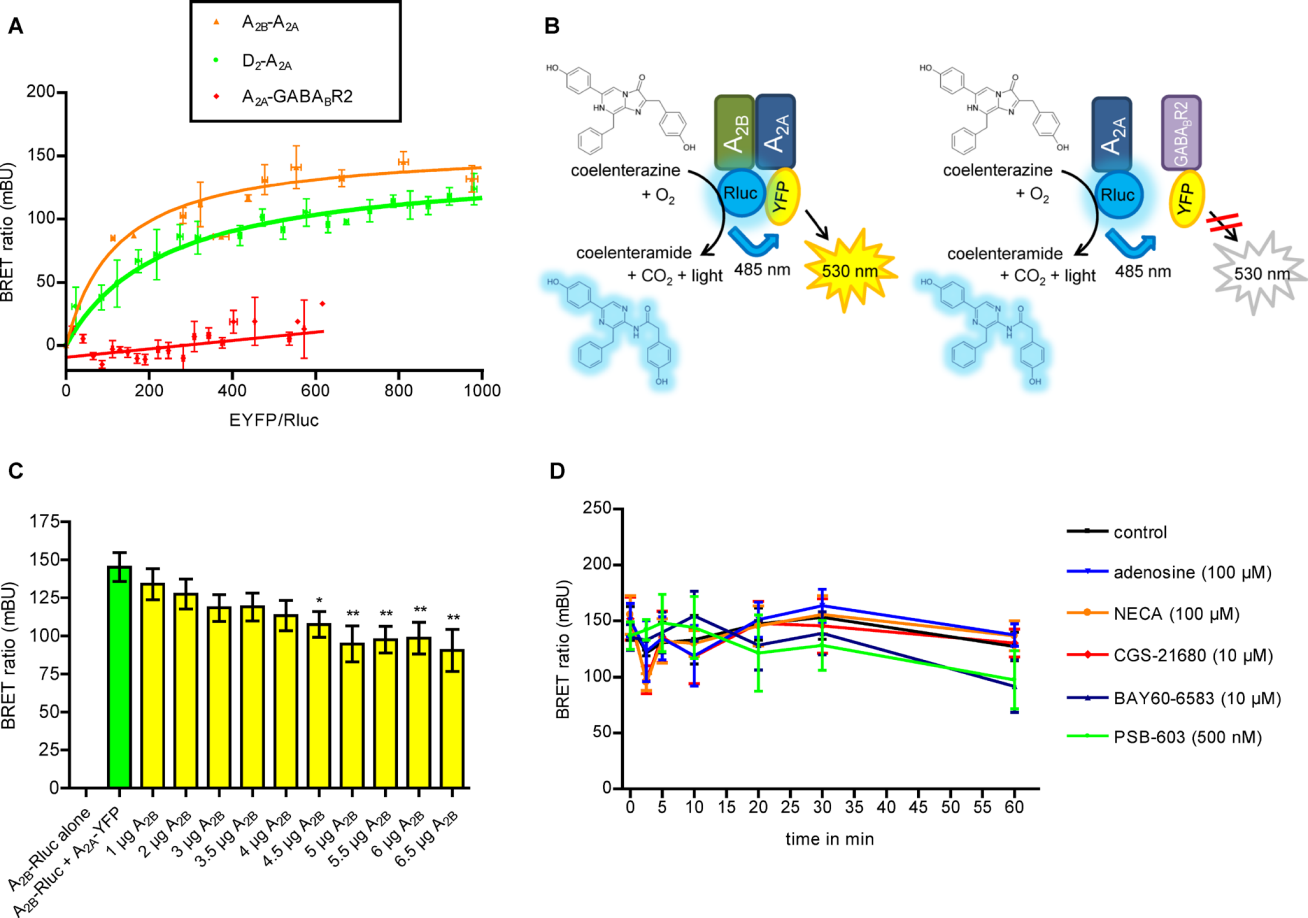
Biophysical assays using A_2A_ and A_2B_ARs fused to BRET donor and acceptor (**A**) BRET saturation curves: CHO-K1 cells were transiently co-transfected with a constant amount of A_2A_, A_2B_ or D_2_ receptors fused to *Rluc* and increasing amounts of cDNA for A_2A_ or GABA_B2_ receptors fused to EYFP. BRET experiments were performed in duplicates for A_2B_-Rluc and A_2A_-YFP (▲) (*n* =11) with a BRET_max_ = 158 ± 10 mBU and BRET_50_ = 122 ± 58, positive control D_2_-Rluc and A_2A_-YFP (●) (*n =* 15) BRET_max_ = 144 ± 6 mBU and BRET_50_ = 239 ± 40, and negative control A_2A_-Rluc and GABA_B2_-YFP (♦) (*n =* 13). (**B**) Schematic representation of the BRET experiments. (**C**) BRET competition experiments were performed (*n =* 4, in triplicates) in cells transfected with 1.25 µg of cDNA for A_2B_-Rluc, 2.5 µg of cDNA for A_2A_-YFP and increasing amounts of cDNA for untagged A_2B_ARs. The one-way ANOVA with Dunnett’s post-hoc test showed a significant decrease in the BRET signal compared to cells which were not transfected with untagged A_2B_ARs (green column; ^*^p < 0.05; ^**^p < 0.01). (**D**) CHO cells were transiently co-transfected with 2 µg of cDNA for A_2B_-Rluc and 3 µg of cDNA for A_2A_-YFP. Different agonists (adenosine, NECA, CGS-21680, BAY60-6583) and antagonists (PSB-603) were added and the BRET signal was measured over a time period of 60 min (*n =* 3, in duplicates).

BRET is another biophysical technique that can be utilized to detect protein-protein interactions by measuring energy transfer from a bioluminescence donor to a fluorescent acceptor [35]. To confirm a direct A_2A_-A_2B_AR interaction, BRET experiments were performed in living CHO-K1 cells transiently expressing fusion proteins consisting of a receptor (A_2A_, A_2B_, D_2_, or GABA_B2_) and Rluc (*Renilla luciferase*) or the fluorescent protein EYFP attached to the C-terminus (Figure 2A–2D). Three kinds of experiments were performed as shown in Figure 2A, 2C and 2D. For BRET saturation curves cells were co-transfected with a constant amount of cDNA for Rluc-receptor constructs and increasing concentrations of cDNAs for EYFP-receptor constructs. As a widely accepted positive control for GPCR dimers, donor/aceptor proteins having the dopamine D_2_R-A_2A_AR were used. The results showed a high BRET signal displaying a hyperbolic curve, with a BRET_50_ value of 239 ± 40 and a BRET_max_ value of 144 ± 6 mBU. As a negative control, donor/acceptor proteins having the A_2A_AR and GABA_B2_ receptor pair were used. The combination of A_2A_ and A_2B_ARs resulted in a specific BRET signal which was even higher than the positive control indicating a specific interaction of both receptors. A BRET_50_ value of 122 ± 6 and a BRET_max_ value of 158 ± 10 mBU were determined (Figure 2A). Subsequently, a BRET displacement study was performed in which increasing amounts of unlabeled A_2B_AR were added to A_2B_-Rluc and A_2A_-YFP receptors. The experiment showed a significant decrease in the BRET signal, which was dependent on the added amount of unlabeled A_2B_AR (Figure 2C), indicating displacement by the unlabeled receptor of the Rluc-tagged A_2B_AR in the heteromer. As a final step, potential effects of A_2A_ and A_2B_AR agonists and antagonists on A_2A_-A_2B_ heteromer formation were studied. The BRET signal after 60 min of treatment with agonists (adenosine, nonselective; NECA, nonselective; CGS-21680, A_2A_-selective; BAY60–6583, A_2B_-selective) or the A_2B_ antagonist PSB-603 was similar to that obtained in the absence of ligands (Figure 2D), thus indicating that A_2A_-A_2B_ heteromer formation was not influenced by those receptor ligands.

These results were further corroborated by BiFC experiments, which provided strong evidence for a very close interaction between A_2A_ and A_2B_ARs (Supplementary Figures 1, 2).

### *In situ* proximity ligation experiments in the rat brain

**Figure 3:**
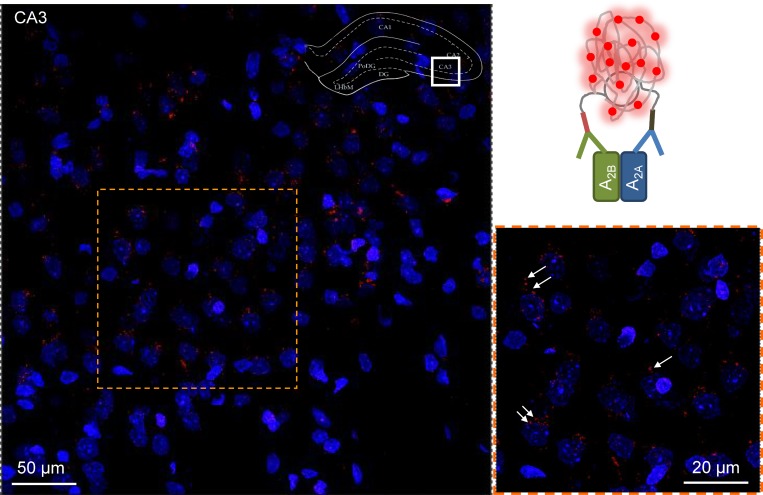
*In situ* proximity ligation assay in rat hippocampus A_2A_-A_2B_AR-specific PLA clusters in the *CA3* region of the *dorsal hippocampus* of the rat (Bregma: –3.6 mm). The sampled region is taken from the framed section of the dorsal hippocampus in the upper right corner of the figure. The microphotographs taken are based on 20 Z-scans (1 µm each). The nuclei are shown in blue. A high density of PLA positive clusters in red are visualized mainly in the pyramidal cell layer shown also in higher amplification in the panel at the lower right part of the figure. A few are indicated by arrows. The diameter range of the clusters is 0.5–2 µm. They are mainly located in a perisomatic position around the blue nuclei but also in the neuropil. A low density of specific PLA clusters is also found in the radiatum and oriens close to the pyramidal cell layer.

The PLA combines the high specificity and affinity of antibodies (PLA probe) with the sensitivity of quantitative polymerase chain reactions (PCR) to detect proteins that are forming molecular complexes in native sources [38]. Initially we studied the recombinant CHO-A_2A_-A_2B_ cell line to investigate the receptors’ proximity (Supplementary Figure 3A–3C) and obtained small, brightly green fluorescent spots each of which represents a single A_2A_-A_2B_AR heteromer (Supplementary Figure 3C). Next we performed *in situ* PLA focusing on the dorsal hippocampus of the rat brain (Figure 3, Supplementary Figures 4, 5) where moderate to high densities of PLA-specific clusters were found. It should be noted that the molecular layer of the dentate gyrus lacked PLA clusters, and the unspecific labeling there was similar to that observed in negative control sections obtained by omitting the primary anti-A_2A_ antibody. Furthermore, few PLA positive clusters were observed in the oriens of the *CA1* areas. In contrast, a high density of A_2A_-A_2B_ specific clusters was found in the *CA3* pyramidal cell layer, mainly in perisomatic location (Figure 3), where PLA-positive clusters had diameters from 0.5–2 µm. They were present also in lower densities in the radiatum and oriens. In all these regions PLA positive clusters were also found in the neuropil. The *CA1* showed a similar distribution pattern (as compared with *CA3*), with high dot/cluster densities within the pyramidal cell layer. An important difference was, however, the diameter range of the clusters, which appeared to be reduced in this *CA1* region versus *CA3* (Supplementary Figure 4).

In the polymorphic layer of the dentate gyrus (PoDG), a high density of specific A_2A_-A_2B_ clusters was observed in both perisomatic and neuropil position. The range of diameter size in the clusters was similar to that in the *CA3* area (Supplementary Figure 5).

### Pharmacological implications of A_2A_-A_2B_AR heteromer formation

To study the pharmacology of A_2A_-A_2B_AR heteromers, native as well as recombinant cell lines were investigated. Expression levels of A_2A_ and A_2B_ARs were analyzed by reverse transcriptase (RT) PCR, Western blot analysis and radioligand binding studies (Supplementary Figure 6A–6E). Recombinant cell lines were prepared to control the proportion of A_2A_ and A_2B_AR expression (A_2B_ ≥ A_2A_, and A_2A_ > A_2B_) (Supplementary Table 1).

### Radioligand-receptor binding studies

**Figure 4:**
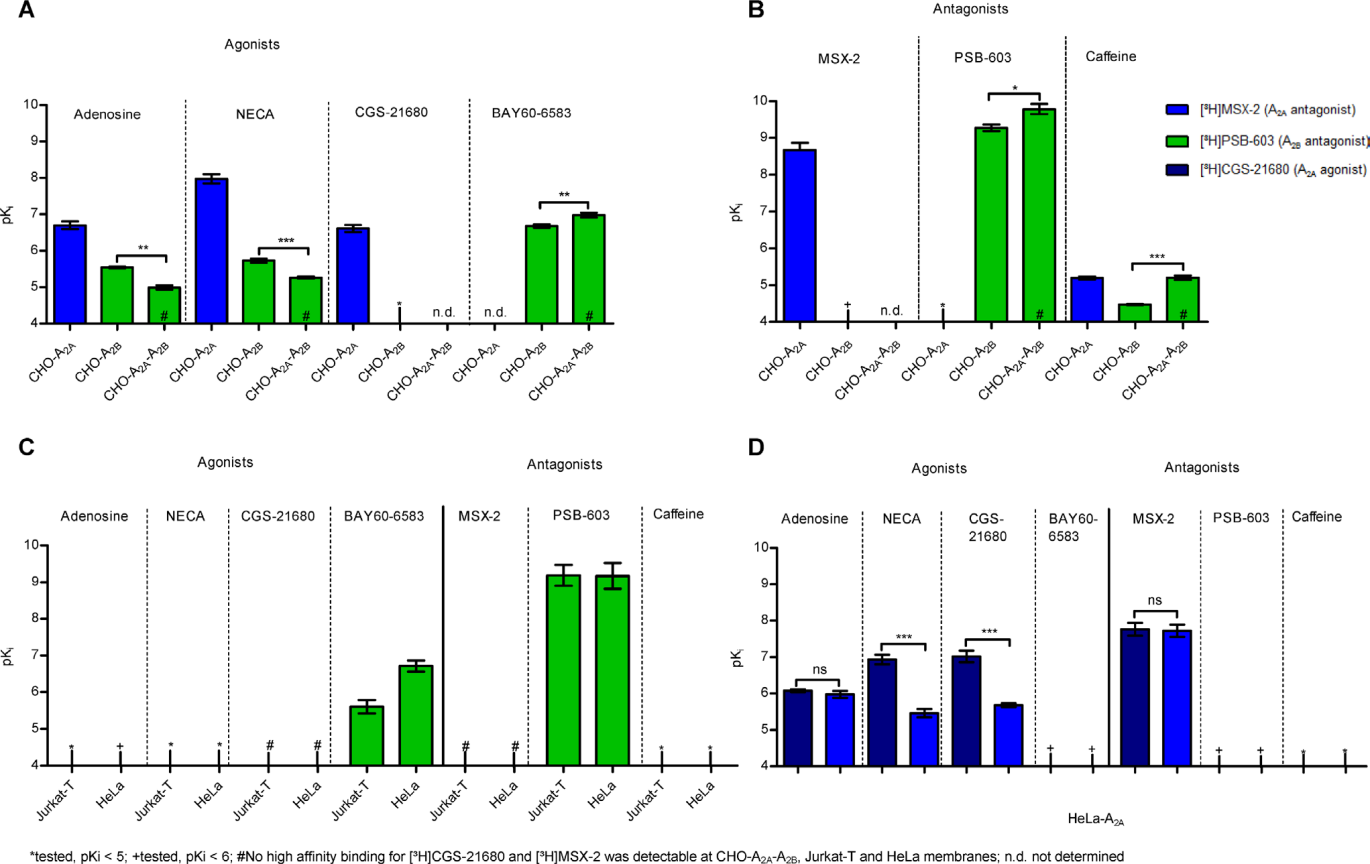
Affinities of AR agonists and antagonists in different cells expressing A_2A_ and A_2B_ARs (**A**) Competition binding experiments of agonists versus 1 nM [³H]MSX-2 at A_2A_-expressing membranes, and versus 0.3 nM [³H]PSB-603 at A_2B_- and at A_2A_-A_2B_AR-expressing membranes of CHO cells. The two-tailed *t*-test showed significant differences. ^***^p < 0.001, ^**^p < 0.01, *n =* 2–3, see also Supplementary Table 3. (**B**) Competition binding experiments of AR antagonists versus 1 nM [³H]MSX-2 at A_2A_-expressing, and versus 0.3 nM [³H]PSB-603 at A_2B_ - and at A_2A_-A_2B_AR co-expressing CHO cell membranes. The two-tailed *t*-test showed significant differences. ^***^p < 0.001, ^*^p < 0.1, *n =* 3, see also Supplementary Table 3. (**C**) Competition binding experiments of AR agonists and antagonists versus 0.3 nM [³H]PSB-603 at Jurkat-T and HeLa cell membranes, *n =* 3–6, see also Supplementary Table 3. (**D**) Competition binding experiments agonists and antagonists versus 5 nM [³H]CGS-21680, and versus 1 nM [³H]MSX2, respectively, at HeLa cell membranes recombinantly overexpressing A_2A_ARs. The two-tailed *t*-test showed significant differences. ns: not significant, ^***^p < 0.001, *n =* 3–4, see also Supplementary Table 3.

Radioligand binding studies were performed using the A_2B_-selective antagonist radioligand [³H]PSB-603 and two A_2A_-selective radioligands, the antagonist [³H]MSX-2, and the agonist [³H]CGS-21680 (Supplementary Figure 7A–7C, Supplementary Table 2). A selective radiolabeled agonist for A_2B_ARs is currently not available. Labeling of A_2B_ARs with [³H]PSB-603 demonstrated high A_2B_ expression in membrane preparations of CHO-A_2B_ and CHO-A_2A_-A_2B_ (A_2B_ ≥ A_2A_) cell lines both of which displayed similar A_2B_ expression levels (502 and 418 fmol/mg protein, respectively). Jurkat-T (220 fmol/mg protein) and HeLa cells (80 fmol/mg protein) had lower A_2B_AR expression levels (Supplementary Figure 6E, Supplementary Table 2). As expected, in cells lacking significant A_2B_ expression (CHO-K1, CHO-HA-A_2A_, HEK-A_2A_), no high-affinity binding of the A_2B_-selective antagonist radioligand [³H]PSB-603 was observed (Supplementary Figure 7C). In cells that co-expressed both receptors (CHO-A_2A_-A_2B_ cells, HeLa cells, Jurkat-T cells, native human T-lymphocytes), specific binding of [³H]PSB-603 was detected, and its affinity was similar to that determined at CHO cells expressing only A_2B_ARs (Supplementary Figure 8A, Supplementary Table 2). Native primary human lymphocytes displayed a moderate expression level of A_2B_ARs, lower to that of A_2A_ARs. Upon activation with phytohemagglutinin (PHA), the A_2B_ expression level remained virtually unaltered (Supplementary Figure 9). All radioligand binding results on A_2B_ARs were in agreement with the data obtained in RT-PCR and Western blot experiments (Supplementary Figure 6A–6D, Supplementary Table 2). The A_2A_-selective radioligands [^3^H]MSX-2 and [^3^H]CGS-21680 labeled A_2A_ARs in CHO-A_2A_ cells and in cells expressing more A_2A_- than A_2B_ARs (Figure 4, Supplementary Figure 7A, 7B, Supplementary Table 2). Native primary human lymphocytes displayed specific binding of [^3^H]MSX-2 indicating A_2A_AR expression, that was significantly upregulated (by about 4-fold) upon activation with PHA (Supplementary Figure 9, Supplementary Table 2). Unexpectedly, in cells with similar or higher expression of A_2B_ as compared to A_2A_ARs no high-affinity binding of either A_2A_-selective radioligand, [^3^H]MSX-2 or [^3^H]CGS-21680, was observed (Supplementary Figure 8A, 8B, Supplementary Table 2). Competition binding assays versus [^3^H]PSB-603 were performed to determine the A_2B_ affinity of selected agonists and antagonists while A_2A_ affinity of compounds was determined versus [^3^H]MSX-2. Indeed, the latter was only possible in cell lines expressing more A_2A_ than A_2B_ARs since high affinity binding of the A_2A_-selective radioligands was abolished when A_2B_ receptors were co-expressed (see above).

CHO-A_2A_ and CHO-A_2B_ cell lines displayed the expected affinities of agonists and antagonists typical for, respectively, A_2A_ or A_2B_ARs (Figure 4A, 4B, Supplementary Table 3). In CHO-A_2A_-A_2B_ cell membranes, which showed a similar or slightly higher expression of A_2B_ than of A_2A_ARs, the agonists adenosine, NECA, and BAY60-6583 and the antagonists PSB-603 and caffeine displayed only slightly modulated A_2B_ affinities (Figure 4A, 4B, Supplementary Table 3). We subsequently studied a T-cell line, namely Jurkat-T cells, which express similar amounts of A_2A_ and A_2B_ARs, the level of which is, however, lower than in the recombinant CHO-A_2A_-A_2B_ cells. The A_2B_-selective radioligand [^3^H]PSB-603 displayed high affinity binding which was displaced by the A_2B_-selective partial agonist BAY60-6583 (Figure 4C, Supplementary Figure 8A, Supplementary Tables 2, 3). However, no high-affinity binding was observed for the A_2A_-selective radioligands [³H]MSX-2 and [³H]CGS-21680 (Supplementary Figure 8A, 8B, Supplementary Table 2), although we could clearly detect A_2A_AR protein expression in Jurkat-T cells (Supplementary Figure 6C). In native T-lymphocytes, isolated from healthy human blood donors, the expression of the A_2A_AR was higher than that of the A_2B_AR (Supplementary Figure 9, Supplementary Table 2). High affinity binding was then observed for the A_2A_-selective radioligand [^3^H]MSX-2 as well as for the A_2B_-selective radioligand [^3^H]PSB-603 (Supplementary Figure 8A, 8B, Supplementary Table 2). In HeLa cells, which natively express more A_2B_ than A_2A_ARs, again, no high-affinity binding was obtained for the A_2A_-selective radioligands [³H]MSX-2 and [³H]CGS-21680 (Supplementary Figure 8A, 8B, Supplementary Table 2) although we could clearly detect the A_2A_AR protein in this cell line (Supplementary Figure 6D). As a next step we overexpressed the human A_2A_AR containing an HA tag in HeLa cells to obtain a cell line which expressed more A_2A_ than A_2B_ARs (Supplementary Figure 6D, Supplementary Table 2). This led to the recovery of high-affinity binding for the A_2A_-selective radioligands (Figure 4D, Supplementary Table 2). NECA and CGS-21680 showed higher affinity versus the agonist radioligand than versus the antagonist radioligand, whereas the antagonist MSX-2 displayed similar affinities versus both radioligands (Figure 4D, Supplementary Table 3), results that are typical for A_2A_ARs [39].

### cAMP accumulation assays

**Figure 5:**
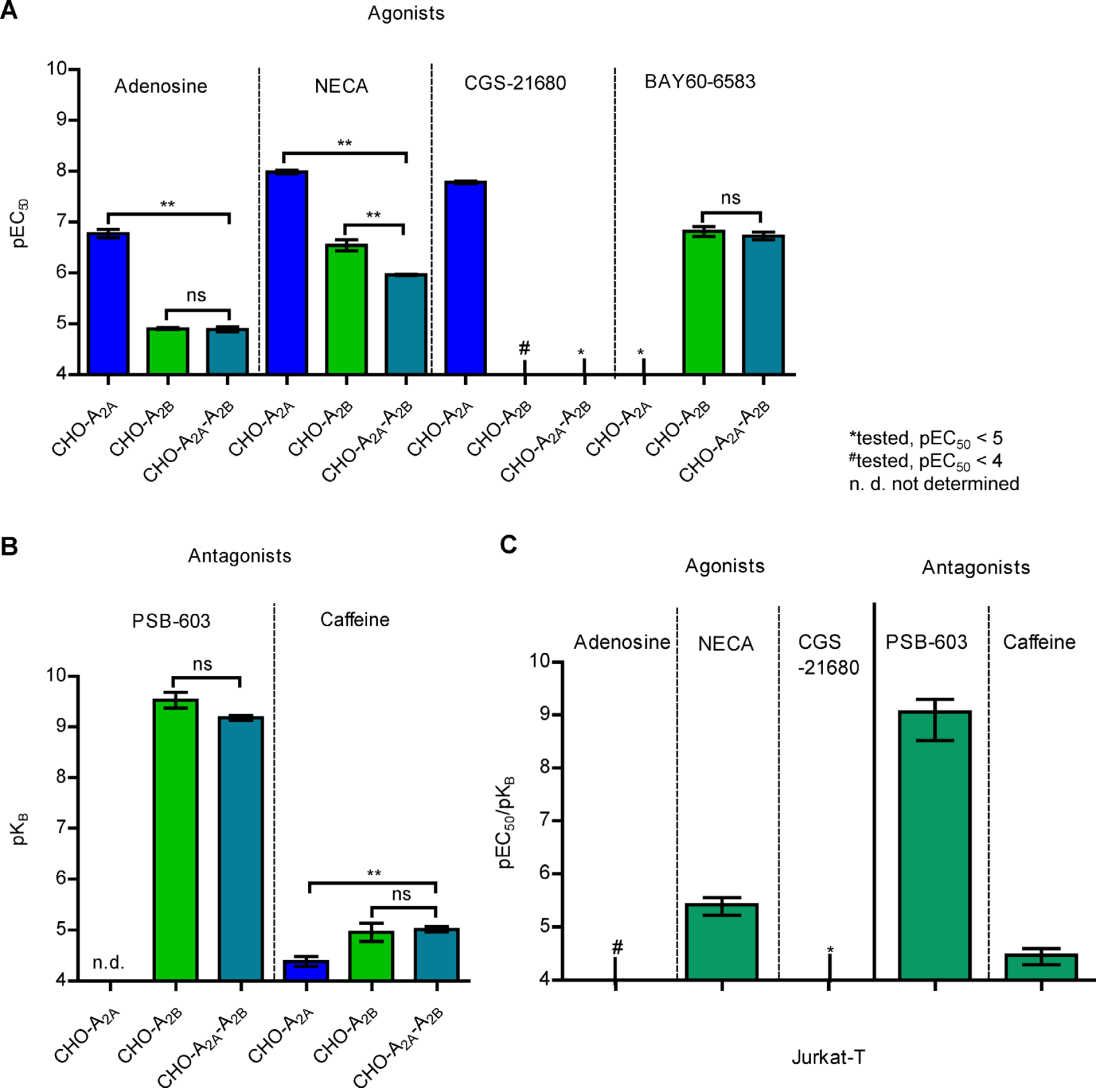
cAMP accumulation in recombinant CHO cell lines and in Jurkat-T cells (**A**) pEC_50_ values of AR agonists in cAMP accumulation assays in CHO-A_2A,_ CHO-A_2B_ and CHO-A_2A_-A_2B_ cells. The one-way ANOVA with Dunnett’s post-hoc test indicated significant differences. ns: not significant, ^**^p < 0.01, *n =* 2–4, see also Supplementary Table 4. The A_2A_AR agonist CGS-21680 showed only a negligible signal in CHO-A_2A_-A_2B_ cells at concentrations of up to 100 µM. (**B**) pK_B_ values for AR antagonists in cAMP accumulation assays in CHO-A_2A_, CHO-A_2B_ and CHO-A_2A_-A_2B_ cells with agonist stimulation by NECA. The one-way ANOVA with Dunnett’s post-hoc test showed significant differences. ns: not significant, ^**^p < 0.01, *n =* 4–6, see also Supplementary Table 4. (**C**) pEC_50_ values of agonists and pK_B_ values of antagonists determined in cAMP accumulation assays at Jurkat-T cells (*n =* 3, see also Supplementary Table 4).

A_2A_ and A_2B_ARs are coupled to G_s_ proteins, activating adenylate cyclase. Thus, cAMP accumulation assays were performed at CHO cells stably expressing the A_2A_AR, the A_2B_AR, or both. Adenosine increased cAMP accumulation with an EC_50_ value of 174 nM in CHO cells expressing the “high affinity” A_2A_AR, and with an EC_50_ value of 12,500 nM in CHO cells expressing the “low-affinity” A_2B_AR. The CHO-A_2A_-A_2B_ cell line co-expressing both subtypes showed virtually the same EC_50_ value (13,100 nM) as the cell line *only* expressing A_2B_ARs (Figure 5A, Supplementary Table 4). The metabolically more stable adenosine analog NECA displayed a similar behaviour. The potent A_2A_-selective agonist CGS-21680 (EC_50_ 16.6 nM in CHO-A_2A_ cells) was inactive in CHO-A_2A_-A_2B_ cells (EC_50_ > 10,000 nM). In contrast, the A_2B_-selective partial agonist BAY60-6583 (EC_50_ 165 nM in A_2B_AR-expressing cells) had similar potency in the A_2A_-A_2B_-coexpressing cell line (EC_50_ 193 nM) (Figure 5A, Supplementary Table 4). Antagonist potencies were determined by measuring concentration-response curves for the non-selective agonist NECA in the presence or absence of different antagonists, and K_B_ values were calculated. The A_2B_-selective antagonist PSB-603 and the non-selective antagonist caffeine showed very similar K_B_-values at CHO-A_2A_-hA_2B_ (PSB-603, K_B_ 0.673 nM; caffeine, K_B_ 9,900 nM) as at CHO-A_2B_ cells (PSB-603, K_B_ 0.358 nM; caffeine, K_B_ 15,600 nM) (Figure 5B, Supplementary Table 4).

We next employed Jurkat-T cells as a native cell line expressing similar amounts of A_2A_ and A_2B_ARs. These cells behaved like the CHO-A_2A_-A_2B_ cell line displaying moderate potency for the physiological agonist adenosine and the structurally related agonist NECA. Again, CGS-21680 was inactive at concentrations up to 10 µM, while the A_2B_-selective antagonist PSB-603 and the nonselective antagonist caffeine displayed K_B_-values in the same range as those determined at CHO-A_2B_ cells (Figure 5C, Supplementary Table 4).

### cAMP assays at HEK-A_2A_ cells transiently transfected with increasing amounts of A_2B_AR

**Figure 6:**
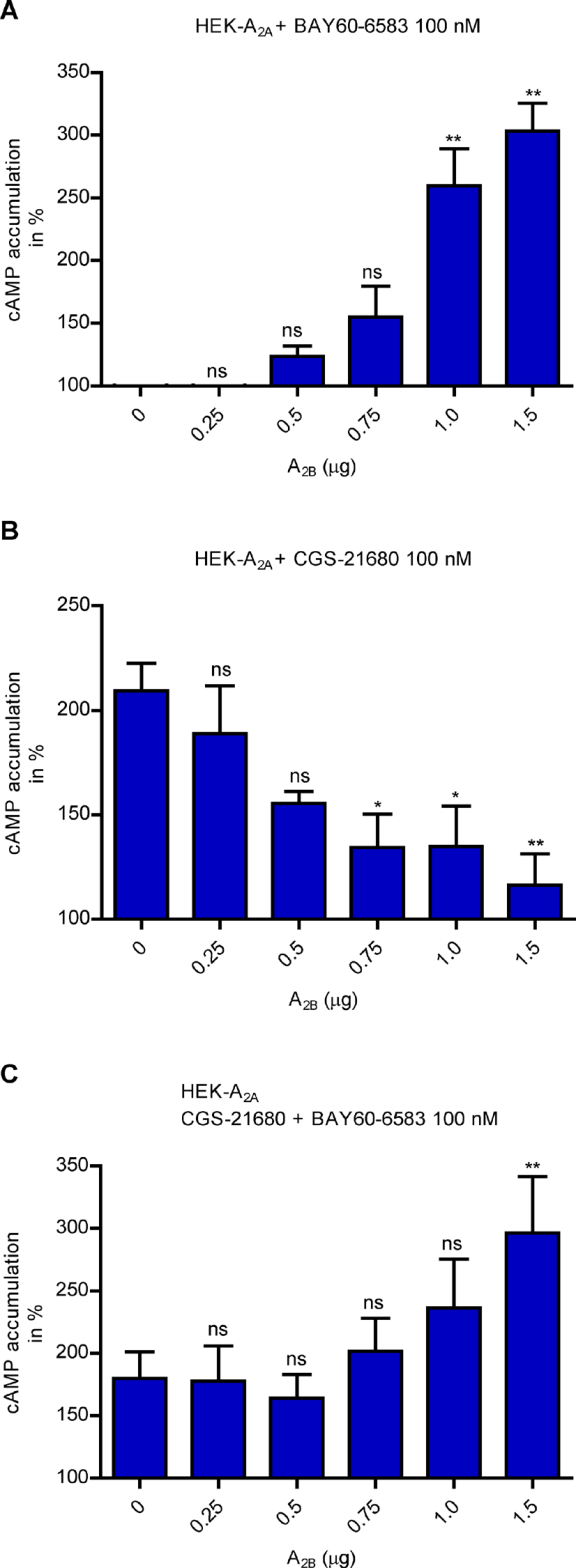
cAMP determination in cells coexpressing variable proportions of A_2A_ and A_2B_ARs (**A**) Stimulation of cAMP accumulation induced by 100 nM of the A_2B_-selective partial agonist BAY60-6583 at HEK-A_2A_ cells transiently transfected with increasing amounts of cDNA for A_2B_AR (0.25–1.5 µg). The basal cAMP level, i.e. HEK-A_2A_ cells only with medium but without the agonist, was set at 100%. The one-way ANOVA with Dunnett’s post-hoc test showed significant differences. ns: not significant, ^**^p < 0.01 (*n =* 2, in triplicates). (**B**) Stimulation of cAMP accumulation induced by 100 nM of the A_2A_-selective agonist CGS-21680 at HEK-A_2A_ cells transiently transfected with increasing amounts of cDNA for A_2B_AR (0.25–1.5 µg). Basal cAMP, i.e. HEK-A_2A_ cells only with medium but without the agonist, was set at 100%. The one-way ANOVA with Dunnett’s post-hoc test showed significant differences. ns: not significant, ^*^p < 0.05, ^**^p < 0.01 (*n =* 2, in triplicates). (**C**) Stimulation of cAMP accumulation induced by a combination of 100 nM of the A_2A_-selective agonist CGS-21680 and of 100 nM of the A_2B_-selective partial agonist BAY60-6583 at HEK-A_2A_ cells transiently transfected with with increasing amounts of cDNA for A_2B_AR (0.25–1.5 µg). Basal cAMP, i.e. HEK-A_2A_ cells only with medium but without the agonist, was set at 100 %. The one-way ANOVA with Dunnett’s post-hoc test showed significant differences. ns: not significant, ^**^p < 0.01 (*n =* 2, in triplicates).

Next, the effect of the expression of different amounts of A_2B_ARs on A_2A_AR pharmacology was studied. To this end human embryonic kidney (HEK293T) cells stably expressing the human A_2A_AR were transfected with human A_2B_AR cDNA to transiently express increasing amounts of A_2B_ARs. The A_2B_-selective agonist BAY60-6583 (100 nM) showed a significant increase in cAMP accumulation with increasing amounts of transiently transfected A_2B_ARs confirming functionality of the A_2B_AR (Figure 6A). In contrast, when stimulating the cells with the A_2A_-selective agonist CGS-21680 (100 nM), a significant decrease in cAMP accumulation with increasing amounts of the transiently transfected A_2B_AR was observed suggesting an inhibition of the A_2A_AR through the A_2B_AR (Figure 6B). Stimulation of the cells with a combination of both receptor subtype-selective agonists (100 nM each), showed a significant increase in cAMP accumulation with increasing amounts of transiently transfected A_2B_AR additionally suggesting a dominant role for the A_2B_AR (Figure 6C).

### Dynamic mass redistribution assays

**Figure 7:**
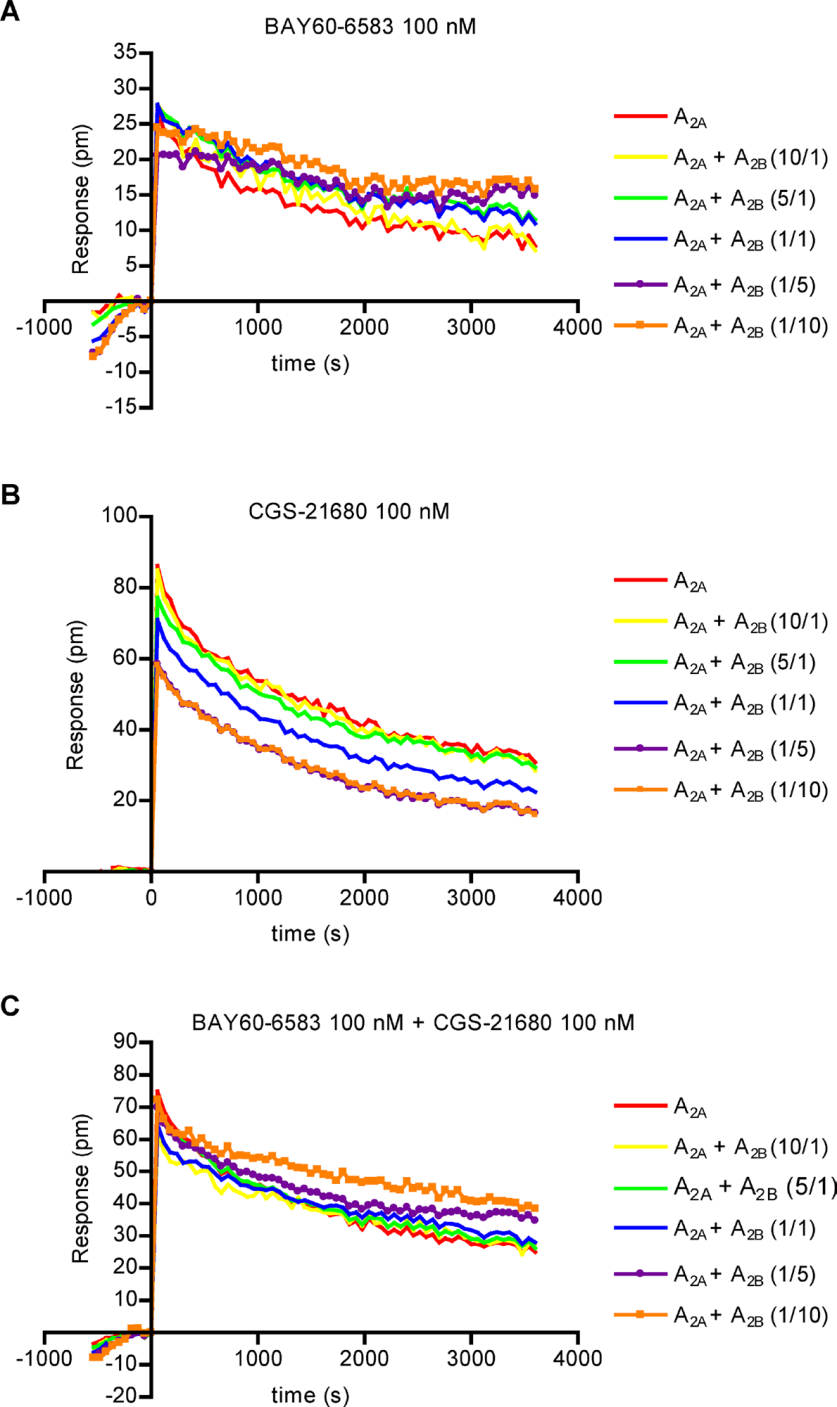
Label-free assays in cells coexpressing variable proportions of A_2A_ and A_2B_ARs (**A**) Time-dependent dynamic mass redistribution (DMR) measurement induced by 100 nM of the A_2B_-selective partial agonist BAY60-6583 in HEK293T cells transiently transfected with a constant amount of cDNA for A_2A_AR and increasing amounts of cDNA for A_2B_AR, or *vice versa*, a constant amount of cDNA for A_2B_AR and increasing amounts of cDNA for A_2A_AR. (**B**) DMR measurement induced by 100 nM of the A_2A_-selective agonist CGS-21680 in HEK293T cells transiently transfected with a constant amount of cDNA for A_2A_AR and increasing amounts of cDNA for A_2B_AR, or *vice versa*, a constant amount of cDNA for A_2B_AR and increasing amounts of cDNA for A_2A_AR. (**C**) DMR measurement induced by a combination of 100 nM of the A_2A_-selective agonist CGS-21680 and of 100 nM of the A_2B_-selective partial agonist BAY60-6583 at HEK293T cells transiently transfected with a constant amount of cDNA for A_2A_AR and increasing amounts of cDNA for A_2B_AR, or *vice versa*, a constant amount of cDNA for A_2B_AR and increasing amounts of cDNA for A_2A_AR.

Finally, dynamic mass redistribution (DMR) assays providing a holistic readout were performed in HEK293T cells transiently transfected with different ratios of A_2A_ and A_2B_AR cDNA. The A_2B_-selective agonist BAY60-6583 (100 nM) displayed a time-dependent increase in the signal with increasing amounts of transiently transfected A_2B_AR confirming the presence of functional A_2B_ARs (Figure 7A). In contrast, when stimulating the cells with the A_2A_-selective agonist, CGS21680 (100 nM), a time-dependent decrease in signal was observed with increasing amounts of transiently transfected A_2B_AR suggesting an inhibition of the A_2A_AR-mediated DMR signal through the A_2B_ARs (Figure 7B). Stimulation of the cells with a combination of both, A_2A_ and A_2B_AR agonist (100 nM each), showed a significant increase in signal with increased amounts of transiently transfected A_2B_AR, but not with increased amounts of A_2A_AR, additionally suggesting within the heteromer a dominant role for the A_2B_AR (Figure 7C).

## DISCUSSION

Adenosine is an important signaling molecule, and the A_2A_ and A_2B_AR subtypes are established (A_2A_) or potential (A_2B_) drug targets. Both receptors are G_s_ protein-coupled, but the A_2B_AR requires much higher adenosine concentrations to be activated than the A_2A_AR, and its physiological role remains enigmatic. In the present study we demonstrate that A_2B_ARs physically interact with A_2A_ARs forming A_2A_-A_2B_ heteromers. This interaction leads to new signalling properties as observed in cAMP experiments at Jurkat-T and further native and recombinant cells. Although A_1_, A_2A_ and A_3_ homomeric ARs and several heteromers of A_1_ and A_2A_ARs had previously been described [1, 40–41], neither homo- nor heteromers of A_2B_ARs have been unambiguously demonstrated so far. Our study was motivated (i) by the fact that A_2A_ and A_2B_ARs are frequently co-expressed; (ii) by the observation that A_2A_AR pharmacology appears to be strikingly different than expected in a number of A_2A_-A_2B_ co-expressing cells and tissues, (iii) by the still unknown physiological role of the “low-affinity” A_2B_ARs; (iv) by contradictory results on pro- or anti-inflammatory effects mediated by the A_2B_AR, and (v) by the observation that A_2A_ and A_2B_ARs upon activation – although they are both G_s_ protein-coupled – often show opposite effects [25]. A_2A_-A_2B_ heteromerization had previously been postulated to be required for high A_2B_AR expression, and the C-terminus of the A_2A_AR has been suggested to play an important role in this respect [42]. However, we could not confirm the published results. In fact, high A_2B_AR expression is observed in many cancer cells including those which express lower amounts of A_2A_ARs [12–13, 43–45]. Nevertheless, co-immunoprecipitation experiments on A_2A_-A_2B_AR-coexpressing HEK293 cells had provided the first indication of a possible existence of A_2A_-A_2B_ heteromers [42]. In the present study, by applying several complementary techniques, we provide compelling evidence that A_2A_ and A_2B_ form heteromeric di-/oligomers in recombinant as well as in native cells and tissues. FRET experiments confirmed specific interaction between both receptor subtypes (Figure 1A). So far the structure of the heteromers and their interaction surface is still unknown. In the well-characterized A_2A_-D_2_ heteromer the long C-terminus of the A_2A_AR is involved in heteromerization [46]. To gain insight into the A_2A_-A_2B_ heteromer interface, we removed the C-terminus of the A_2A_AR and examined the resulting truncated receptor mutant co-expressed with the A_2B_AR in FRET experiments. The observed FRET signal was comparable to the one obtained for the wt A_2A_-A_2B_AR heteromer (Figure 1A, 1B) indicating that the C-terminus is not involved in the formation of A_2A_-A_2B_ heteromers. Extensive BRET studies, including displacement experiments with untagged receptors, showed high, specific BRET signals for the A_2A_-A_2B_AR pair (Figure 2A, 2C). Incubation of cells with various agonists, adenosine (nonselective), NECA (nonselective), CGS-21680 (A_2A_-selective), BAY60-6583 (A_2B_-selective), or antagonists (PSB-603, A_2B_-selective) did not lead to a significant change of the signal (Figure 2D). This means that A_2A_-A_2B_ heteromer formation is independent of the presence of A_2A_ or A_2B_AR ligands and stable in the presence of ligands. Furthermore BiFC experiments in which part of the EYFP sequence is attached to the A_2A_, the other part to the A_2B_AR, resulted in positive signals. These data convincingly underscore that both receptors are directly interacting, and that an A_2A_-A_2B_ heterodi- or -oligomer must have been formed (Supplementary Figures 1, 2). A_2A_-A_2B_ heteromer formation in CHO-A_2A_-A_2B_ cells was confirmed by yet another technique, proximity ligation assays (PLA), using specific modified primary antibodies. The positive PLA signals obtained in living CHO-A_2A_-A_2B_ cells, but not in non-transfected CHO cells, indicated close proximity of both receptors in the CHO cell line that coexpress them (Supplementary Figure 3). Thus, all conducted biophysical and biochemical techniques, namely FRET, BRET, BiFC and PLA studies, provided evidence that A_2A_ and A_2B_ARs can form heteromers in living cells. Importantly, the existence of A_2A_-A_2B_ heteromers in native tissues was confirmed by using the PLA approach (Figure 3, Supplementary Figures 4, 5) thus proving their physiological relevance. Remarkably, heteromers were detected in sections from hippocampus of the rat. Receptor pharmacology was altered in cells expressing the two receptors and, therefore, also expressing A_2A_-A_2B_ heteromers, as compared to that in cells expressing only one receptor subtype. This was initially observed in a recombinant system investigating CHO-A_2A_, CHO-A_2B_ and CHO-A_2A_-A_2B_ cells, the latter of which had higher A_2B_ than A_2A_AR expression as assessed by RT-PCR and Western blot analysis. In cell lines only expressing A_2A_ or A_2B_ARs, radioligand binding studies provided the expected affinities for standard ligands, i.e. high affinity binding of the selective A_2A_AR ligands CGS-21680 (agonist) and MSX-2 (antagonist), but lacking affinity for the A_2B_-selective ligands BAY60-6583 (partial agonist) and PSB-603 (antagonist) at the CHO-A_2A_ cells and *vice versa* for the CHO-A_2B_ cells (Supplementary Tables 2, 3). At the CHO-A_2A_-A_2B_ cell line, however, no binding for the A_2A_-selective radioligands [³H]MSX-2 and [³H]CGS-21680 was detected although we could clearly prove both the presence of A_2A_AR mRNA and A_2A_AR protein in this cell line (Supplementary Figures 8A, 8B, 6A, 6B, Supplementary Table 2) and its cell surface localization (Supplementary Figure 3C). The A_2A_-A_2B_ heteromer behaved almost like an A_2B_AR by displaying low affinity for adenosine and NECA (Figure 4A, 4B), high affinity for PSB-603 and no significant sign of A_2A_AR activation. cAMP accumulation assays yielded results in line with the pharmacology obtained in binding studies (Figure 5A–5C, Supplementary Table 4). Moreover in cAMP assays as well as in DMR assays at HEK-A_2A_ cells transiently transfected with increasing amounts of the A_2B_AR (Figure 6A–6C, Figure 7A–7C) a clear inhibiton of A_2A_AR function through A_2B_ARs could be demonstrated. This means that the A_2A_AR signaling appears to be completely blocked in the A_2A_-A_2B_ heteromer. On a molecular level, this would mean that the A_2B_AR modulates the conformation of the binding site of the A_2A_AR in such a way that it either loses its affinity for ligands completely, and only the A_2B_ binding site is available for interaction with ligands, or the A_2A_ binding site in the heteromer switches to A_2B_-like properties. Both would in principle be possible. In fact, the binding sites for adenosine in A_2A_ and A_2B_ARs are almost identical differing only in a single amino acid (A_2A_: L249, A_2B_: V250), and yet their affinity for adenosine is very different. It is therefore likely that the ligand binding site of A_2A_ and A_2B_ARs is readily amenable to allosteric modulation. The discovery of A_2A_AR action blockade by the A_2B_AR was very consistent when assayed in heterologous expression systems, in cell lines or in primary cells. Other groups had previously confirmed A_2A_ and A_2B_ mRNA expression in Jurkat-T cells (A_2B_ ≥ A_2A_) and discovered that the selective A_2A_AR agonist CGS-21680 was nearly inactive in cAMP assays [47], and also the binding of [³H]CGS-21680 (12.5 nM) to Jurkat-T cell membranes was found to be negligible [48]. These results can now be explained by taking into account our discovery that the A_2A_AR is non-functional within the A_2A_-A_2B_ heteromer context present in Jurkat-T cells. Our findings also provide a mechanistic basis to interpret previous results, namely the altered AR pharmacology in cells expressing both A_2B_ and A_2A_ARs with similar or higher levels of A_2B_ (A_2B_ ≥ A_2A_). In Supplementary Table 5 we have collected the appropriate literature data that may now be interpreted in the light of our main finding. For example, in the human bladder carcinoma cell line T24 the expression of A_2B_ and A_2A_ receptors was demonstrated by RT-PCR (A_2B_ > A_2A_). In cAMP assays NECA and adenosine were able to induce cAMP accumulation whereas the A_2A_-selective agonist CGS-21680 was inactive [45]. Wei *et al.* showed expression of all four AR subtypes in three prostate cancer cell lines by qRT-PCR and Western blot experiments, with domination of the A_2B_AR (A_2B_ > A_2A_). NECA and the selective A_2B_AR agonist BAY60-6583, but not the A_2A_-selective agonist CGS-21680, concentration-dependently induced cAMP accumulation [13]. Recently, Hajiahmadi *et al*. demonstrated expression of all four AR subtypes in three human ovarian cancer cell lines by qRT-PCR and Western blot, in which, again, the A_2B_AR was the most abundant one (A_2B_ > A_2A_), and NECA but not the A_2A_AR agonist CGS-21680, concentration-dependently induced cAMP accumulation [12]. In contrast, in A_2A_-A_2B_-co-expressing cell lines and tissues in which the expression level of A_2A_ARs is higher than that of A_2B_ (A_2A_ > A_2B_), e.g. in T-lymphocytes from human blood, PC12 cells, or HMC-1 cells, canonical A_2A_ and A_2B_ pharmacology is observed (Supplementary Tables 2, 5) [8, 16, 49–50]. It is likely that A_2A_-A_2B_AR heteromers exist in these cells but in a proportion that does not prohibit activation of *free* A_2A_ARs (that cannot be blocked by A_2B_ARs). Thus, regulation of A_2A_ and A_2B_ expression levels can in turn govern the pharmacological outcome of stimulation with adenosine or synthetic AR agonists. Both G_s_-coupled A_2_AR subtypes play important (patho)physiologic roles and are co-expressed on many cell types and tissues [6, 8, 11, 51]. Up-regulation of A_2A_AR expression in different tissues can be found under various pathological conditions. For example, lymphocytes from amyotrophic lateral sclerosis (ALS) or multiple sclerosis (MS) patients show increased A_2A_AR levels [16–17]. NF-kappaB was found to enhance hypoxia-driven T-cell immunosuppression via activation of upregulated A_2A_ARs [52]. In a study on isolated perfused mouse heart investigating the contributions of A_2A_ and A_2B_ARs on cardiac flow, the A_2A_-selective agonist CGS-21680 and the non-selective agonist NECA increased coronary flow in A_2B_ knockout (KO) mice to a significantly higher degree than in wildtype (WT) mice [51]. The A_2A_-selective antagonist SCH58261 blocked NECA-induced increase in coronary flow to a higher degree in KO than in WT mice. The authors explained these discrepancies by an observed upregulation of A_2A_ARs (ca. 20% as estimated by Western blot) in mesenteric arterioles of KO as compared to WT mice. However, an additional or alternative explanation could be that by deletion of A_2B_ARs, heteromer formation would no longer be possible leading to a de-blocking of the A_2A_ARs and thus a higher number of free receptors with typical A_2A_ pharmacology. Several studies have led to the proposal that GPCR heteromers may constitute drug targets in their own right and that heteromers can be upregulated in disease [53]. It appears that A_2A_ and A_2B_ARs display very high affinity for each other, and whenever they are co-expressed they would form stable heteromers with a distinct pharmacology. This implies that the A_2A_-A_2B_ interface and the interactions must be unique to produce such a remarkable and persistent disappearance of A_2A_AR ligand recognition and signaling through allosteric receptor-receptor modulation.

## MATERIALS AND METHODS

### Molecular biology

For FRET experiments the cDNAs of the human A_2A_AR and the C-terminal truncated mutant A_2A_1-293R were amplified by PCR using primers that delete the stop codon of the receptors and introduce *EcoRI* and *AgeI* restriction enzyme sites. The resulting PCR products were cloned in-frame with *EcoRI/AgeI* into the vectors pEYFP-N1 and pGFP^2^-N3, respectively. The cDNA of the human A_2B_AR was also cloned with *EcoRI/AgeI* into the vector pGFP^2^-N3. For a positive control a fusion protein GFP^2^-EYFP was used. The cDNA of GFP^2^ was amplified by PCR using primers that delete the stop codon of the protein and introduce *BamHI* and *AgeI* restriction enzyme sites. The resulting PCR product was cloned in-frame with *BamHI/AgeI* into pEYFP-N1 plasmid, respectively. The cDNA of the GABA_B_R2-EYFP receptor construct was a gift from Prof. Franco. For BRET experiments the cDNAs of the human A_2A_ and A_2B_AR were amplified by PCR using primers that delete the stop codon of the receptors and introduce *EcoRI* and *BamHI* restriction enzyme sites. The resulting A_2A_ and A_2B_AR PCR products were cloned in-frame with *EcoRI/BamHI* into pRluc-N2. The dopamine D_2_-pRluc-N2 receptor construct was a gift from Prof. Franco. The A_2A_AR cDNA was cloned in-frame with *EcoRI/AgeI* into pEYFP-N1, respectively. For BRET experiments *Rluc*-EYFP was used as a positive control, and *Rluc* alone and EYFP plasmids alone were employed as negative controls. For the positive control the cDNA of *Rluc* was amplified by PCR using primers that delete the stop codon of the protein and introduce *EcoRI* and *BamHI* restriction sites. The resulting PCR product was cloned in-frame with *EcoRI/BamHI* into pEYFP-N1 plasmid, respectively. For BRET displacement experiments, the cDNA of the human A_2B_AR was cloned with *EcoRI/NotI* into the pcDNA3.1(+) plasmid.

Transiently transfected HEK293T cells were prepared by subcloning the cDNAs of the human A_2A_ and A_2B_ AR with *HindIII/BamHI* into the vector pcDNA3.1(+). All restriction enzymes and supplements for the molecular biology were obtained at NEB (Frankfurt, Germany). DNA and RNA purification kits were obtained at Zymo Research (Freiburg, Germany) or Life Technologies GmbH (Darmstadt, Germany).

### Transient transfection of CHO-K1 cells for FRET and BRET experiments

For FRET and BRET experiments CHO-K1 cells were transiently co-transfected with constant amounts of the receptor-donor DNA (e.g. A_2B_-GFP^2^ or A_2B_-*Rluc*) and constant or increasing amounts of receptor-acceptor DNA (e.g. A_2A_-EYFP) using Lipofectamine 2000 (Thermo Fisher Scientific, Waltham, USA). Moreover the receptor-donor DNAs and receptor-acceptor DNAs were transfected alone to determine their contribution to the detection channels (spectral signature). The cells were harvested 24 h after transfection and used for FRET, and BRET experiments, respectively.

### FRET experiments analyzed by fluorimetry

FRET experiments were conducted as previously described [36]. 20 µg of the transfected cells (100 µl) were distributed in duplicates in a black 96-well plate with black bottom for fluorescence measurements. The fluorescence signals were detected by a Mithras LB 940 fluorimeter using a 10-nm bandwidth excitation filter at 405 nm and 500 nm and 10-nm bandwidth and 25-nm bandwidth emission filters corresponding to 510 nm and 535 nm (GFP^2^ exication 405 nm, emission 510 nm; EYFP exication 500 nm, emission 535 nm). Background fluorescence signals from non-transfected CHO-K1 cells were substracted. For all experiments the gain settings and the read time of 0.1 s were kept identical.

### Quantification of FRET signal

Quantification of the FRET signals was performed as described by Elder *et al.* according to a sensitized emission method [54]. This method requires correction for donor bleed-through (donor fluorophore emission into the acceptor channel) and acceptor cross-excitation (direct excitation of acceptor fluorophores by donor excitation). For that, the contributions of GFP^2^ and EYFP proteins alone to the two detection channels were measured in experiments with cells expressing only one of these proteins. The spectral signatures of the different receptors fused to either GFP^2^ or EYFP did not significantly vary from the determined spectral signatures of the fluorescent proteins alone. The donor bleed-through correction factor (DER, measured in cells expressing only donor fluorophors) and acceptor cross-excitation factor (AER, measured in cells expressing only acceptor fluorophores) were calculated using the following formula [54]:

$$AER=\frac{I^{DA}}{I^{AA}}\;DER=\frac{I^{DA}}{I^{DD}}$$

The AER is the ratio of emission into the acceptor channel when using donor excitation relative to when using acceptor excitation. The DER is the ratio of emission into the acceptor channel relative to emission into the donor channel, when using donor excitation [54]. The corrected FRET signal was then calculated according to the following formula [54]:

$$cFRET=I^{DA}-DER*I^{DD}-AER*I^{AA}$$

Different FRET normalisation equations can be used to determine the efficiency of the FRET signal. Here we used the *N*_FRET_ normalizing method according to Shyu *et al.*, which takes changes in donor and acceptor concentrations into account [55]:

$$N_{FRET}=\frac{cFRET}{\sqrt{\left(I^{DD}*I^{AA}\right)}}$$

FRET signal analyses were done in Excel and results were displayed using GraphPad Prism 4.

### Bioluminescence resonance energy transfer (BRET) experiments analyzed by fluorimetry

BRET experiments were conducted as previously described [36]. 20 µg of the transfected cells (100 µl) were distributed in duplicates into a black 96-well plate with black bottom for fluorescence and a white 96-well plate with white bottom for bioluminescence measurements. Signals were detected by a Mithras LB 940 fluorimeter calculating the integration for bioluminescence using filters for 440–500 nm (485 nm maximum emission of bioluminescence), and for fluorescence with a filter for 510–590 nm (530 nm maximum emission of EYFP). To confirm equal expression of Rluc and increasing expression of EYFP, for each sample bioluminescence and fluorescence was measured before starting the experiment. EYFP fluorescence was defined as the fluorescence of the sample minus the fluorescence of cells expressing only *Rluc*-tagged receptors. For BRET, 5 µM coelenterazine-H (PJK, Kleinblittersdorf, Germany) was added to the samples and measurements were performed after 1 min (net BRET determination) and after 10 min (*Rluc* luminescence quantification). Net BRET was defined as the bioluminescence of the sample minus the bioluminescence of cells expressing only *Rluc*-tagged receptors. BRET signals were determined by calculating the ratio of the light emitted by EYFP over the light emitted by the *Rluc A*. milliBRET unit (mBU) is the BRET ratio × 1000. Curves were fitted using nonlinear regression. Displacement studies were performed in triplicates at a constant BRET ratio, around the BRET_50_ of the A_2B_AR-Rluc/A_2A_AR-YFP pair, and increasing amounts of the A_2B_AR. For testing the effects of compounds on heteromerization of A_2A_AR and A_2B_AR the agonists adenosine (100 µM), NECA (100 µM), CGS-21680 (A_2A_R, 10 µM), BAY60-6583 (A_2B_R, 10 µM), and the antagonist PSB-603 (A_2B_R, 500 nM) were added for 60 min to cells which expressed A_2B_AR-Rluc and A_2A_AR-YFP, around the BRET_50_ value. The final DMSO concentration in these experiments was 2.5 % and experiments were performed in triplicates.

### *In situ* proximity ligation assay in the rat brain

The experiments were carried out in accordance with the European Directive 2010/63/EU and were approved by the Bioethical Committee at Karolinska Institutet. Male Sprague-Dawley (derived from the licensed animal breeder Charles River, Sulzfeld, Germany), weighing between 260–310 g at the beginning of the experiment, were used. The animals (*n* = 5) were housed individually in standard plastic rodent cages (25 cm × 30 cm × 30 cm) in a colony room maintained at 21 ± 1° C and 40–50 % humidity under a 12-hour light-dark cycle (lights on at 6:00 am). Rodent food and water were available *ad libitum*. All animals used for the *in situ* PLA neurochemical study were experimentally naive.

To study the distribution of the adenosine A_2A_-A_2B_ heteroreceptor complex in the rat brain the *in situ* proximity ligation assay (*in situ* PLA) was performed as described previously [56].

### Retroviral transfection

CHO cells stably expressing HA-tagged human A_2A_ or A_2B_ARs, or both human A_2A_ and A_2B_ARs were generated using a retroviral transfection system [5]. HeLa cells stably overexpressing HA-tagged human A_2A_ARs were also generated using a retroviral transfection system [5]. For generating the A_2A_-A_2B_ co-expressing cell line, GP^+^env AM12 cells were co-transfected with 6.75 µg of the pQCXIP-A_2A_ vector construct and 3.25 µg of VSV-G using Lipofectamine 2000. The supernatant medium containing the modified virus was filtered and transferred into a small flask of 70% confluent CHO-A_2B_ cells. After infection the selection of the CHO-A_2A_-A_2B_ cells was started after 48 h by the addition of 10 µg/ml of puromycin.

### Cell culture

Chinese hamster ovary cells (CHO-K1), GP^+^ env AM12 cells and CHO cells stably expressing human A_2A_ARs, HA-tagged human A_2A_AR or human A_2B_AR were cultured as drecribed previously [5, 57]. CHO cells stably co-transfected with human A_2A_AR and A_2B_AR were maintained in Dulbecco's Modified Eagle Medium (DMEM-F12) medium supplemented with 10% (v/v) fetal calf serum (FCS), 100 units/ml penicillin, 100 µg/ml streptomycin, 800 µg/ml G418 and 10 µg/ml puromycin at 37° C and 5 % CO_2._ Human Jurkat-T cells natively expressing A_2A_ and A_2B_ARs were cultured in RPMI 1640 medium supplemented with 10 % (v/v) fetal calf serum (FCS), 100 units/ml penicillin, 100 µg/ml streptomycin and 2 mM *L*-glutamine. HeLa cells natively expressing human A_2A_ and A_2B_ARs were cultured in DMEM supplemented with 10% (v/v) FCS, 100 units/ml penicillin, 100 µg/ml streptomycin. HeLa cells stably overexpressing the human HA-tagged A_2A_AR were cultured in the same medium with the addition of 800 µg/ml of G418. HEK293T cells were grown in DMEM supplemented with 2 mM *L*-glutamine, 5% (v/v) FCS, 100 units/ml penicillin, 100 µg/ml streptomycin, and minimal essential medium non-essential amino acid solution (1:100) at 37° C in an atmosphere of 5 % CO_2_. All cell culture media and supplements were obtained from (Darmstadt, Germany), Sigma-Aldrich (Munich, Germany) or Applichem (Darmstadt, Germany).

### Membrane preparation

The preparations of CHO and HeLa membranes recombinantly (or natively) expressing human A_2A_ and/or A_2B_AR subtypes were performed as previously described [5]. Membranes from HEK-A_2B_ or A_2A_ cells, which were used for some of the radioligand competition binding studies, were purchased from Perkin Elmer (Waltham, USA). The preparation of Jurkat-T cell membranes was performed as follows: a Jurkat-T cell suspension was centrifuged in 50 ml falcon tubes at 200 g, 4° C, 5 min. The supernatant was discarded and the cell pellets were quickly frozen at –80° C. The defrosted pellets were then resuspended in ice-cold 25 mM Tris-buffer, 0.32 M sucrose, 1 mM ethylenediaminetetraacetic acid (EDTA), 0.1 mM phenylmethanesulfonylfluoride (PMSF), pH 7.4, and homogenized with an Ultra-Turrax (30 s on ice). The homogenate was centrifuged for 10 min at 1000 *g* at 4° C. The resulting pellets were discarded and the supernatant was centrifuged for 1 h at 48.000 g at 4° C. Pellets containing the membranes were resuspended in aqua bidest., homogenized with an Ultra-Turrax (30 s on ice) and centrifuged under the same conditions. The supernantant was discarded and the pellets were resuspended in 50 mM Tris-buffer, pH 7.4 and homogenized with a glass Teflon homogenizer on ice. The resuspended and homogenzied pellets were aliquoted and stored at –80° C until use. The protein content of all membrane preparations was determined with the Lowry method [58].

### Radioligand binding assays

Radioligand receptor binding experiments at membrane preparation of native (HeLa) and recombinant cells (CHO- or HEK cells recombinantly expressing A_2A_ and/or A_2B_ARs) using the A_2B_-selective antagonist radioligand [³H]PSB-603 (spec. activity: 79 Ci/mmol) to detect A_2B_ARs were performed as previously described [59]. Competition binding experiments at various membrane preparations of native (HeLa, Jurkat-T) and recombinant cells (CHO or HEK cells expressing A_2A_ and/or A_2B_ARs) using the A_2A_-selective antagonist radioligand [^3^H]MSX-2 (spec. activity: 85 Ci/mmol) to detect A_2A_ARs were performed in analogy to described procedures [39]. The assay was performed in a final volume of 400 µl containing 4 µl of test compound dissolved in DMSO, 196 µl buffer (50 mM Tris-HCl, pH 7.4), 100 µl of radioligand solution in the same buffer (1 nM), and 100 µl of membrane preparation (10 to 200 µg protein per vial, 2 U/ml adenosine deaminase (ADA) 20 min incubation at rt). Non-specific binding was determined in the presence of unlabeled MSX-2, or CGS-15943, respectively (10 µM), both giving identical results.

Competition binding experiments at membrane preparations of native cells (HeLa, Jurkat-T cells) or recombinant cells (CHO, HEK or HeLa recombinantly expressing the human A_2A_AR) using the agonist radioligand [^3^H]CGS-21680 (spec. activity: 39 Ci/mmol) were performed in analogy to described procedures [5]. The assay was performed in a final volume of 400 µl containing 4 µl of test compound dissolved in DMSO, 196 µl buffer (50 mM Tris-HCl, 10 mM MgCl_2_ pH 7.4), 100 µl of radioligand solution in the same buffer (5 nM), and 100 µl of membrane preparation (10 to 200 µg of protein per vial, 2 U/ml ADA 20 min incubation at rt). Non-specific binding was determined in the presence of NECA (10 or 50 µM, respectively) or CGS-15942 (final concentration 10 µM); all gave identical results. All data were analyzed with GraphPad Prism, Version 4 (GraphPad Inc., La Jolla, CA).

### Radioligand binding assays at Jurkat-T cell membranes

Competition binding experiments at Jurkat-T cell membrane preparations using the A_2B_-selective antagonist radioligand [³H]PSB-603 to detect A_2B_ARs were performed in a final volume of 1000 µl containing 25 µl of test compound dissolved in DMSO/50 mM Tris-buffer pH 7.4 (1:1), 375 µl of assay buffer (50 mM Tris-HCl, pH 7.4), 100 µl of radioligand solution in the same buffer (final concentration 0.3 nM), and 500 µl of membrane suspension (100–200 µg protein per vial, 2 U/ml 20 min incubation at rt). Non-specific binding was determined in the presence of unlabeled PSB-603 (10 nM) or DPCPX (10 µM); both gave similar results. After an incubation time of 60 min at rt, the assay mixture was filtered through GF/B glass fiber filters. Washing buffer: 50 mM Tris-HCl buffer, 0.1% bovine serum albumin (BSA), pH 7.4.

### cAMP assays

The determination of cAMP accumulation in recombinant CHO and in Jurkat-T cells was performed as previously described [5, 60]. K_B_-values for the A_2A_-specific antagonist MSX-2, the A_2B_-specific antagonist PSB-603 and the non-selective antagonist caffeine were determined versus the full agonist NECA. K_B_-values were calculated using the child equation [61].

### Transient transfection of HEK293T cells for cAMP assays

HEK293T cells were transiently transfected with the corresponding cDNA (pcDNA3.1+-hA_2B_, pcDNA3.1+-hA_2A_) by the polyethylenimine (Sigma) method [62]. The determination of AR-induced cAMP accumulation in HEK293T cells transiently expressing A_2A_ and A_2B_ARs was performed as previously described [62].

### Dynamic mass redistribution assays

Dynamic mass redistribution (DMR) at HEK293T cells transfected with A_2A_ and A_2B_ARs was determined as described previously [62]. In brief, 24 h before the assay, cells were seeded at a density of 7,500 cells per well in 384-well sensor microplates with 40 µl of growth medium and cultured for 24 h (37° C, 5 % CO_2_) to obtain 70–80% confluent monolayers. Prior to the assay, cells were washed twice with assay buffer (Hank’s balanced salt solution; HBSS with 20 mM HEPES, pH 7.15) and incubated for 2 h in 40 µl per well of assay buffer in the DMR reader at 24° C. Hereafter, the sensor plate was scanned and a baseline optical signature was recorded before adding 10 µl of test compound dissolved in assay buffer containing 0.1% DMSO and DMR responses were monitored for at least 8,000 s using an EnSpire^®^ Multimode Plate Reader (PerkinElmer, Waltham, MA, USA) by a label-free technology. Data were analyzed using EnSpire Workstation Software v 4.10.

## SUPPLEMENTARY MATERIALS FIGURES AND TABLES





## Abbreviations

ADA: adenosine deaminase
AR: adenosine receptor(s)
BAY60-6583: 2-({6-amino-3,5-dicyano-4-[4-(cyclopropylmethoxy)phenyl]pyridin-2-yl}sulfanylacetamide
BiFC: bimolecular fluorescence complementation
BRET: bioluminescence resonance energy transfer
BSA: bovine serum albumin
cDNA: complementary deoxyribonucleic acid
CGS-21680: (2-*p*-[2-carboxyethyl]phenethylamino)-5´-*N*-ethylcarboxamidoadenosine
CHO: Chinese hamster ovary
DAPI: 4′,6-diamidin-2-phenylindole
DEPC: diethylpyrocarbonate
DMEM: Dulbecco’s Modified Eagle Medium
DMSO: dimethylsulfoxide
DPCPX: 8-cyclopentyl-1,3-dipropylxanthine
DMR: dynamic mass redistribution
EDTA: ethylenediamine tetraacetic acid
EGTA: ethyleneglycoltetraacetic acid
EYFP: enhanced yellow fluorescent protein
FCS: fetal calf serum
FRET: Förster resonance energy transfer
GFP^2^: green fluorescent protein variant 2
GPCR: G protein-coupled receptor
G418: geneticin
HA: human influenza hemagglutinin tag
h: human
HBSS: Hank’s balanced salt solution
KO: knock-out
KRH: Krebs-Ringer-Hepes
mRNA: messenger ribonucleic acid
MSX-2: 3-(3-hydroxypropyl)-7-methyl-8-(*m*-methoxystyryl)-1-propargylxanthine
NECA: 5′-*N*-ethylcarboxamidoadenosine
PBS: phosphate buffered saline
PEI: polyethylenemine
PMSF: phenylmethanesulfonyl fluoride
PVDF: polyvinylidene fluoride
PSB-603: 8-(4-(4-(4-chlorophenyl)-piperazine-1-sulfonyl)phenyl)-1-propylxanthine
Rluc: Renilla Luciferase
RNA: ribonucleic acid
Ro 20-1724: 4-(3-butoxy-4-methoxyphenyl)methyl-2-imidazolidone
RPMI: Roswell Park Memorial Institute
rt: room temperature
RT-PCR: reverse transcriptase-polymerase chain reaction
TBS: Tris buffered saline
Tris: Tris(hydroxymethyl)aminomethan
