# Supplementary material for: Adenosine A2A receptor ligand recognition and signaling is blocked by A2B receptors
Source: Oncotarget. 2018 Feb 6;9(17):13593–611. doi: 10.18632/oncotarget.24423 (PMC5862601; doi:10.18632/oncotarget.24423)
Supplement: Supplementary file 1 [file oncotarget-09-13593-s001.pdf]

Adenosine A_{2A} receptor ligand recognition and signaling is blocked by A_{2B} receptors

SUPPLEMENTARY MATERIALS

Molecular biology

For BiFC experiments the cDNAs of the NYFP (1-155) and CYFP (156-239) were cloned into the pcDNA 3.1(-)plasmid using *EcoRI* and *BamHI* as restriction enzymes. After that the cDNAs of the adenosine A_{2A} and A_{2B}AR, and for the negative control the $GABA_BR2$ were amplified by PCR using primers that delete the stop codon of the receptor and introduce restriction enzyme sites at the same time. The resulting PCR products were cloned in-frame with *NotI/EcoRI* into the pcDNA3.1(-)-NYFP and pcDNA3.1(-)-CYFP plasmids, respectively. Using complementary primers with *XbaI* and *NotI* restrictions sites, a HA-tag (human influenza haemagglutinin) was additionally introduced into the pcDNA3-1(-)-A_{2A}-CYFP constructs. For the positive assay control the cDNAs of the pBiFC-HA-bFosYC155 and pBiFC-bJunYN155, which were a gift from Prof. Dr. Tom Kerppola (Michigan, USA), were used [1].

Transient transfection of CHO-K1 cells for BiFC experiments

For BiFC experiments CHO-K1 cells were transiently transfected with constant amounts of the receptor NYFP constructs (e.g. A_{2B}-NYFP) and increasing amounts of receptor CYFP constructs (e.g. HA-A_{2A}-CYFP). Non-transfected CHO-K1 cells were used in all different experiments for background measurements, and signals were substracted from the obtained data. Cells were transferred from 175 cm^2 flask to 6-well plates (700.000 cells per well) 24 h before transfection and incubated in medium without antibiotics. For transfection (90% confluent) Lipofectamine transfection reagent 2000 was utilized following the product protocol. The ratio of DNA (µg) : Lipofectamine (µl) was 1:1. After an incubation time of 4.5 h the medium was replaced and cells were cultured in 2 ml of DMEM-F12 medium supplemented with 10% (v/v) FCS, 100 units/ml penicillin and 100 µg/ml streptomycin at 37° C in an atmosphere of 5% CO_2. The cells were harvested 24 h after transfection and used for BiFC experiments.

Bimolecular complementation experiments analyzed by fluorimetry

CHO cells were transfected, and after 24 h they were washed twice with 1 ml HBSS buffer containing 10 mM glucose, resuspended in 250 µl HBSS puffer, detached and transferred into 1.5 ml Eppendorf tubes. The protein concentration of all samples was determined using Bradford method. Samples were then diluted to a concentration of 200 µg/ml and 20 µg of the cells (100 µl) were distributed in duplicates in a black 96-well plate with black bottom for fluorescence measurement. EYFP fluorescence was detected by fluorimetry using a 10-nm bandwidth excitation filter at 500 nm and 25-nm bandwidth emission filters corresponding to 535 nm. For all experiments the gain settings were kept identical. For the positive control the pBiFC-HA-bFosYC155 and pBiFC-bJunYN155 were co-transfected. EYFP signal analyses were done in excel (subtraction of background from non-transfected CHO-K1 cells) and results were shown using GraphPad Prism 4. The expression of all receptor constructs was confirmed by immunoblotting.

Immunoblotting analysis of transfected CHO cells used in BiFC experiments

Immunoblotting was performed according to the analysis of Jurkat-T cell membranes and CHO-A_{2A}-hA_{2B} cell membranes. Total extracts from the transiently transfected cells were sonicated in 1-fold sample buffer (0.0625 M Tris-HCl buffer, pH 6.8, 2% SDS, 10% glycerol, 0.1 M DTT, 0.01% bromophenol blue) and diluted to a concentration of 1 µg/µl. All samples were heated at 40° C for 10 min. Proteins (25 µg of all co-transfected receptor samples and 10 µg of co-transfected transcription factors) were separated by SDS-PAGE, transferred to *polyvinylidene fluoride* (PVDF) membranes and analyzed by immunoblotting using specific antibodies. To control the protein transfer to the blotting membrane Ponceau S staining was performed. The membranes were incubated for 2 min in 0.2% Ponceau S staining solution and then rinsed with distilled water. For Western blot analysis monoclonal antibodies anti-GFP (incubation 90 min at room temperature, 1:3000, Covance, Denver, USA, MMS-118P) for detecting NYFP receptor constructs only, and anti-HA (incubation 90 min rt, 1:1000, Covance, Denver, USA, MMS-101P) for detecting HA-CYFP receptor constructs, were used as the first antibody. Anti-mouse horseradish peroxidase was used as the secondary antibody (incubation 50 min rt, 1:3000 horseradish peroxidase conjugated α-rabbit antibody, Jackson Immuno

Research Laboratories, West Grove, PA, USA). The immunoreactive bands were visualized using the enhanced chemiluminescence system from Pierce (now Thermo Fisher, Waltham, USA).

Preparation of the proximity ligation assay probes for the *in situ* proximity ligation assay at recombinant CHO-A_{2A}-A_{2B} cells

The PLA probes which were needed to perform the in situ proximity ligation assays using the commercial Duolink in situ Proximity Ligation Assay system from Olink Bioscience were obtained by modifying two primary antibodies. For that the commercial Duolink in situ Probemaker Kits was used (PLUS and MINUS, both Olink Bioscience, Uppsala, Sweden). The anti-A_{2A} antibody (ARP59952_P050 from Aviva Systems Biology; San Diego, CA, USA,) was modified using the Duolink in situ Probemaker PLUS Kit; and the anti-A_{2B} antibody (AAR-003, Alomone Labs, Jerusalem, Israel) was modified using the Duolink in situ Probemaker MINUS Kit following the manufacturer's instructions. Prior to the modification step, the antibodies were purified to remove any disturbing agents using protein G magnetic beads (Thermo Fisher Scientific, Waltham, USA). Subsequently, the buffers were exchanged using desalting columns (Pierce Polyacrylamide Spin Desalting Columns 7k MWCO, Thermo Fisher Scientific, Waltham, MA, USA). The purified antibody samples were concentrated using centrifugal filter devices with a molecular weight cutoff of 100 kDa (Amicon Ultra-4 100k, Merck Millipore, Billerica, MA, USA). The purified antibody samples with a concentration of 1 μg/μL were ready to be modified.

In situ proximity ligation assay at recombinant CHO-A_{2A}-A_{2B} cells

We used a rolling circle amplification (RCA) proximity ligation assay kit. In the case of close proximity of the A_{2A} and A_{2B}AR the two connectors hybridize to the oligonucleotides that are attached to specific A_{2A} and A_{2B}AR primary antibodies. The connector oligonucleotides can then be ligated to form a circularized single-stranded DNA template which is subsequently amplified by PCR. As a result a cluster of single-stranded DNA is formed which can be detected with small fluorescent-labeled oligonucleotides that hybridize to complementary sequences of the amplified template. The fluorecent spots can be observed by confocal laser scanning microscopy. The in situ proximity ligation assay experiments were carried out using the commercial Duolink in situ Detection Reagents Green (Olink Bioscience, Uppsala, Sweden). Two days before the experiment, cells were seeded on coverslips (12 mm). The coverslips were sterilized with ethanol; afterwards, the coverslips were placed into 6-well plates (Sarstedt, Nuembrecht, Germany). Per well 50,000 cells were seeded using 3 ml of the eligible selection medium. The cells were incubated at 37° C, at 5% CO_2 and 95% humidity. At the day of the proximity ligation experiment, the medium was removed and the cells were washed twice with PBS buffer. Then, the fixation step followed using a PBS solution supplemented with 4% paraformaldehyde; the cells were incubated 15 min at rt. Subsequently, the cells were washed 3 times with PBS buffer. Then, the cells were incubated for 20 min at room temperature with a 25 mM glycine solution to reduce autofluorescence, and subsequently washed twice with PBS buffer. The following permeabilization step (using a 0.1% (v/v) Triton X-100 solution) was left out since the used primary antibodies are directed against extracellular epitopes; hence, they do not need to cross the cell membrane; therefore, the blocking step followed to reduce unspecific binding of the antibodies; hence, the cells were incubated for 30 min at room temperature in a PBS solution supplemented with 1% (w/v) BSA. After blocking, the coverslips were removed from the cavities of the 6-well plates and placed on parafilm in a humidity chamber. Preceding this step, the blocking solution was removed from the coverslips by gently tapping the coverslips on a clean piece of tissue paper. Subsequently, the actual in-situ proximity ligation reaction using the Duolink in situ Detection Reagents Green Kit was performed following the manufacturer's instructions. After the proximity ligation reaction the coverslips were incubated with PBS buffer supplemented with 4′,6-diamidino-2-phenylindole (DAPI), 1:10,000) for 2 min at room temperature. Subsequently, the coverslips were washed twice with PBS buffer (2 × 2 min). Finally, the coverslips were mounted on glass slides using ProLong Gold antifade reagent (Thermo Fisher Scientific, Waltham, MA, USA). After the mounting medium had solidified, the coverslips were sealed using nail polish. Fluorescence images were acquired on a Nikon confocal laser scanning microscope A1+ with Ti-Eclipse system (Nikon, Chiyoda, Japan). A 60 × oil objective (Carl Zeiss, Oberkochen, Germany) was used for acquiring high magnification images (with zoom when needed). Furthermore, high resolution images were acquired as z-stack with a 0.2 μm z-interval. All fluorescence images were analyzed using the NIS-Elements software from Nikon.

RNA isolation

RNA was extracted from collected CHO-K1 and CHO-A_{2A}-A_{2B} cells with Trizol reagent (Life Technologies, Darmstadt, Germany). Cells (90% confluent) in 75 cm² cell flasks were washed with 5 ml of phosphate-buffered saline (PBS) and cells were lyzed by adding 3 ml of Trizol reagent. After 10 min of incubation at room temperature, the cell suspension was transferred into 1.5 ml Eppendorf tubes, and 0.2 ml of chloroform per 1 ml of TRIZOL reagent was added. Samples were vortexed for 15 s

and incubated at rt for 10 min. After that, samples were centrifuged at 12,000 *g* for 15 min at 4° C. The mixture separated into a lower red, phenol-chloroform phase, an interphase, and a colorless upper aqueous phase which contained the RNA. The aqueous phase was carefully transferred into new 1.5 ml Eppendorf tubes and 0.5 ml of isopropanol per 1 ml of TRIZOL reagent was added. Samples were mixed, incubated at rt for 10 min and centrifuged at 12,000 *g* for 10 min at 4° C. The supernatant was removed and the RNA pellets were resuspended in 1 ml of 70% aqueous ethanol. Samples were vortexed and centrifuged at 7,500 *g* for 5 min at 4° C. Then, the residual ethanol was removed and RNA pellets were air-dried for 10 min at rt. RNA pellets from each sample were dissolved in 25 µl of diethylpyrocarbonate- (DEPC-) treated water and stored at –20° C.

Reverse transcription PCR

The cDNA was synthesized using a reverse transcription kit (Omniscript RT, Quiagen, Hilden, Germany). Messenger RNA (mRNA), 15 µl from each RNA pool, was transcribed into single-stranded cDNA using oligo dT primers (Qiagen, Hilden, Germany) that specifically hybridize to the poly A-tail of mRNAs. For PCR analyses 2 µl of each cDNA pool were used as a template. cDNA-specific primers for A_{2A}ARs, A_{2B}ARs and beta-actin were used to detect fragments of the receptor genes inside the cDNA pools of CHO-K1 and CHO-A_{2A}-A_{2B} cells. Receptor fragments were amplified using Biomix Red (Bioline, Luckenwalde, Germany). RT-PCR efficiencies were evaluated using primers for mouse/rat beta-actin. Primer sets for the following genes (sense and antisense sequence, respectively) were used: human A_{2A}AR: f-5′-aggatgtggtccccatgaac-3′, human A_{2A}AR: r-5′-ctctccgtcactgccatgag-3′ human A_{2B}AR: f-5′-gaggggtcattgctgtcctc-3′, human A_{2B}AR: r-5′-ggccatattc attgcccact-3, f-β-actin-mouse/rat: 5′-ccctaaggccaaccgtg aaaagat-3′, r-β-actin-mice/rat: 5′-aggtcccggccag ccaggtc-3′. The amplification products (25 µl) were analyzed on 1% agarose gel stained with gelred (Biotium, USA).

Western blot analysis of Jurkat-T cells, HeLa cells, transfected HeLa cells overexpressing A_{2A} ARs and CHO cells co-expressing A_{2A} and A_{2B}ARs

Membrane preparations were used as samples for Western blots. Therefore, liquid homogenization of cells was performed using a Potter-Elvehjem homogenizer [2]. The membrane preparations were subsequently obtained by differential-velocity centrifugation [3]. To remove whole cells and nuclei the homogenate was first centrifuged at 1000 *g* for 10 min. Subsequently, the pellet was discarded and the supernatant was centrifuged at 37,000 *g* for 1 h using an ultra-centrifuge in order to obtain membrane fractions. The protein concentrations were determined by the Bradford method [4]. The samples were separated by SDS-PAGE (10%, approximately 1 h at 200 V). For the anti-A_{2A} immunoblot experiments 25 µg of the CHO-K1 sample, 25 µg of the CHO-A_{2A}-A_{2B} sample, 50 µg of the Jurkat-T lymphocyte sample, 50 µg of the HeLa cell sample, and 50 µg of the HeLa-A_{2A} cell membrane sample were applied. For the anti-A_{2B} immunoblot experiments 10 µg of the CHO-K1, 10 µg of the CHO-A_{2A}-A_{2B} and 10 µg of the Jurkat-T lymphocyte samples were used. For HeLa and HeLa-A_{2A} cells 50 µg were used. Before applying them to the gel the samples were pretreated with heat (80° C) for 10 min. After separation by SDS-PAGE the proteins were electroblotted to nitrocellulose membranes (1 h at 100 V). Subsequently, the blotting was controlled by staining the membranes with a Ponceau solution (0.2% Ponceau S, 5% acetic acid) [7]. Then, the membranes were blocked for at least 1 h at room temperature in phosphate buffered saline (PBS) with 0.1% Tween-20 and 5% dry milk powder. After blocking, the membranes were incubated with the primary antibody overnight at 4° C. The primary antibodies were dissolved in the blocking solution (anti-A_{2A} antibody, AAR-002, 1 : 2000); (anti-A_{2B} antibody, AAR-003, 1 : 1000), both obtained from Alomone Labs, Jerusalem, ISR. For HeLa and HeLa-A_{2A} membrane samples the primary antibodies were used in a different dilution: anti-A_{2A} antibody 1: 1000; anti-A_{2B} antibody 1: 500. After washing three times (3 × 15 min) with PBS containing 0.1% Tween-20 the membranes were incubated with the secondary antibody dissolved in the blocking solution (1:4000 horseradish peroxidase conjugated anti-rabbit antibody (obtained from the Jackson ImmunoResearch Laboratories, West Grove, PA, USA) for 1 h at room temperature. Subsequently, the membranes were washed three times (3 × 15 min) with PBS containing 0.1% Tween-20. The immunoreactive bands were visualized using an enhanced chemiluminescence (ECL) system (SuperSignal West Pico Chemiluminescent Substrate; Thermo Fisher Scientific, Waltham, MA, USA).

Radioligand binding assays at primary human lymphocytes

For competition binding experiments at intact primary human lymphocytes cells were centrifuged in 50 ml Falcon tubes at 200 g, 4° C, and 5 min. The supernatant was discarded and the cell pellet was resuspended in 10 ml of Krebs-Ringer-Hepes- (KRH-) buffer (118 mM NaCl, 4.84 mM KCl, 1.2 mM KH_2PO_4, 2.44 mM $CaCl_2$, 2.43 mM $MgSO_4$, 10 mM HEPES, pH 7.4) 37° C containing ADA (1 U/ml). The cell suspension was incubated for 30 min at 37° C and then centrifuged again at 200 g, 4° C, for 5 min. The supernatant was discarded and the cell pellet was resuspended in KRH-buffer (37° C, 1 U/ml ADA). After another 30 min of incubation at 37° C, the cell

suspension was used for competition binding experiments which were conducted at 37° C.

Competition binding experiments at intact primary human lypmphcytes with the A_1AR antagonist radioligand [^{3}H]DPCPX were performed in a final volume of 500 µl containing 10 µl of test compound dissolved in DMSO/KRH-buffer pH 7.4 (1:1), 290 µl of KRH-buffer (37° C, pH 7.4), 100 µl of radioligand solution in the same buffer (final concentration 5 nM), and 100 µl of cell suspension (5×10^7–1×10^8 cells per 24-vial rack, 1 U/ml ADA). Non-specific binding was determined in the presence of unlabeled DPCPX (final concentration 10 µM). After an incubation time of 60 min at 37° C, the assay mixture was filtered through GF/B glass fiber filters. Harvesting (washing buffer: KRH-buffer, pH 7.4), liquid scintillation counting and data analysis were conducted as described above. Competition binding experiments at intact primary human lymphocytes with the A_3AR antagonist radioligand [^{3}H]PSB-11 (final concentration 1 nM) were performed as described above. Non-specific binding was determined in the presence of unlabeled PSB-10 (final concentration 50 µM). After an incubation time of 45 min at 37° C, the assay mixture was filtered through GF/B glass fiber filters. Harvesting (washing puffer: KRH-buffer, pH 7.4), liquid scintillation counting and data analysis were conducted as described above. Competition binding experiments at intact primary human lymphocytes with the A_{2A} AR antagonist radioligand [^{3}H]MSX-2 were performed in a final volume of 500 µl containing 10 µl of test compound dissolved in DMSO/KRH-buffer pH 7.4 (1:1), 290 µl buffer (KRH-buffer, 37° C, pH 7.4), 100 µl of radioligand solution in the same buffer (final concentration 5 nM), and 100 µl of cell suspension (5×10^7–1×10^8 cells per 24-vial rack, 1 U/ml ADA). Non-specific binding was determined in the presence of unlabeled MSX-2 (final concentration 10 µM). After an incubation time of 30 min at 37° C, the assay mixture was filtered through GF/B glass fiber filters which were previously incubated in 0.3 % aqueous polyethylenemine (PEI) solution for 30 min. Harvesting (washing puffer KRH-buffer, pH 7.4), liquid scintillation counting and data analysis were conducted as described above.

Competition binding experiments at intact primary human lymphocytes with the A_{2B}-antagonist radioligand [^{3}H]PSB-603 (final concentration 0.2 nM), were performed as described above. Non-specific binding was determined in the presence of unlabeled PSB-1115 (final concentration 50 µM). After incubation time of 45 min at 37° C the assay mixture was filtered through GF/B glass fiber filters. Harvesting (washing puffer: KRH-buffer/0.15 % BSA, pH 7.4), liquid scintillation counting and data analysis were conducted as described above. K_D and B_{max} values at intact cells (binding sites per cell) were determined in homologous competition binding experiments.

RESULTS

Bimolecular complementation experiments (BiFC)

To provide further evidence for a very close interaction of protomers in the heteromer bimolecular complementation experiments (BiFC) were performed [5]. The principle of the BiFC assay is to tag both of the receptor proteins with a different, complementary fragment of a fluorescent protein and allow them to form a functional fluorescent protein upon dimerization. The N- and C-terminal fragments of EYFP were tethered to the C-terminal ends of the GPCRs (A_{2A}, A_{2B}, or $GABA_{B2}$). The constructs were transiently expressed in CHO-K1 cells and EYFP fluorescence was measured. Moreover, different flexible linker sequences of 10-22 amino acids between the receptors and the truncated YFP-tags were engineered to optimize complementation. Additionally, a hemagglutinin- (HA-) tag was introduced at the N-terminus of the receptor-CYFP constructs. Different receptor-NYFP and HA-receptor-CYFP linker sequence combinations were assessed for their complementation efficiency. The expression of all receptor constructs was confirmed by Western blots (NYFP: anti-GFP antibody, CYFP: anti-HA antibody see Supplementary Methods). As an internal positive control, the previously described interaction between Jun and Fos transcription factors was determined [63], showing high EYFP fluorescence even at low amounts of DNA used for transient co-expression (Supplementary Figure 1). As a negative control, a constant amount of cDNA for $GABA_{B2}$-NYFP was co-transfected with increasing amounts of cDNA for HA-A_{2A}-CYFP or *vice versa*. This led to a small fluorescence signal which was hardly dependent on the amount of protein expression (Supplementary Figure 1).

In a first control experiment, a constant amount of A_{2A}-NYFP cDNA and increasing quantities of HA-A_{2A}-CYFP cDNA were used for transient transfection. A strong fluorescence signal was already observed at the lowest amount of HA-A_{2A}-CYFP, which further increased with higher transfection loads indicating the formation of A_{2A}-A_{2A}AR homomers (Supplementary Figure 2A). Finally, cells expressing A_{2B}-NYFP were co-transfected with increasing amounts of cDNA for HA-A_{2A}-CYFP. This led to a significant increase in fluorescence, which was saturable upon increasing HA-A_{2A}-CYFP expression levels (Supplementary Figure 2B). These results clearly demonstrated that A_{2A} and A_{2B}ARs form heteromers in recombinant cells. The expression levels of all receptors and transcription factors were confirmed by immunoblots (Supplementary Figures 1, 2A, 2B). The employed methods are, however, not applicable to native, non-transfected cells. For heteromer detection in natural sources we used the proximity ligation assays (PLA) approach, which was first tested in the heterologous expression system.

In situ proximity ligation experiments at recombinant CHO-A_{2A}-A_{2B} cells

The PLA combines the high specificity and affinity of antibodies (PLA probe) with the sensitivity of quantitative polymerase chain reactions (PCR) to detect proteins that are forming molecular complexes in native sources [6]. Initially we studied the recombinant CHO-A_{2A}-A_{2B} cell line to investigate the receptors' proximity. Non-transfected CHO cells were used as a negative control (Supplementary Figure 3A) and, in a further (technical) negative control, CHO-A_{2A}-A_{2B} cells were employed but avoiding ligation reaction (Supplementary Figure 3B). Both negative controls exhibited only little background fluorescence. When using CHO-A_{2A}-A_{2B} cells, apart from sparse background fluorescence (similar to that in the negative controls) small, brightly green fluorescent spots appeared, each of which represents a single A_{2A}-A_{2B}AR heteromer (Supplementary Figure 3C). These results provide further evidence for the close proximity of A_{2A} and A_{2B}ARs in CHO cells co-expressing both AR subtypes and suggest that the PLA method may be used to detect heteromers in native samples.

Cell lines

To study the pharmacology of A_{2A}-A_{2B}AR heteromers, native as well as recombinant cell lines were investigated. CHO-K1 cells were stably transfected with both the A_{2A} and A_{2B}ARs to investigate potential A_{2A}-A_{2B}AR heteromer formation with possibly altered pharmacology. A recombinant cell line which showed a similar expression level of A_{2B} as compared to A_{2A}ARs was selected for further studies. CHO-K1 cell lines only expressing one receptor, A_{2A} or A_{2B}, were also generated for comparison. Moreover, native cells which co-express A_{2A} and A_{2B}ARs were investigated, including human T-lymphocytes, Jurkat-T cells (a human T-lymphocyte-derived immortal cell line that is frequently used as a model for leukemia), and HeLa cells (a human epithelial cell line derived from cervical cancer). In addition, we recombinantly overexpressed the A_{2A}AR in HeLa cells to create a cell line with higher expression of A_{2A} than A_{2B}ARs. RT-PCR experiments using the same RNA and primer concentrations indicated that both AR subtypes were detected at the transcript level in the CHO-A_{2A}-A_{2B} cell line ($A_{2B} > A_{2A}$, Supplementary Figure 6A). In the non-transfected CHO-K1 cells, only beta-actin was detected (Supplementary Figure 6A), which confirms the lack of native A_{2A} and A_{2B}ARs in this cell line [8] as well as the specificity of the designed primers. A_{2A} and A_{2B}AR expression was also detected on the protein level using immunoblot analysis: CHO-K1 cell membranes did not express A_{2A} or A_{2B}ARs, whereas CHO-A_{2A}-A_{2B} co-expressed both A_{2A} and A_{2B}ARs (Supplementary Figure 6B). The protein amount of the A_{2B}AR appeared to be similar or slightly higher than that of the A_{2A}AR, according to the estimated intensity of the band and the different protein amounts (A_{2B}: 10 µg, A_{2A}: 25 µg) on the blot (Supplementary Figure 6B). Jurkat-T, HeLa, and HeLa-A_{2A} cell membranes co-expressed both A_{2A} and A_{2B}ARs (Supplementary Figure 6C, 6D). The expression level of both AR subtypes was moderate in Jurkat-T cell membranes, as indicated by Western blot (Supplementary Figure 6C). The protein amount of the A_{2B}AR appeared to be higher than that of the A_{2A}AR, according to the estimated intensity of the band and the different protein amounts (A_{2B}: 10 µg, A_{2A}: 50 µg) on the blot (Supplementary Figure 6C). We had previously shown by RT-PCR experiments that the Jurkat-T cell line used in our experiments expressed similar amounts of A_1, A_{2A}, and A_{2B}AR mRNA, while the expression of A_3AR mRNA was significantly lower [9]. In contrast, intact primary human lymphocytes were found to express high A_{2A}AR levels and a somewhat lower amount of A_{2B}ARs [9]. HeLa cells have previously been reported to express mRNA for all four AR subtypes, and A_{2B}AR expression was shown to be significantly higher than that of A_{2A}ARs [10]. We confirmed expression of both, A_{2A} and A_{2B}ARs, on the protein level. The protein amount of the A_{2B}AR appeared to be higher than that of the A_{2A}AR, based on the estimated intensity of the band with the same protein amounts (A_{2B}: 50 µg, A_{2A}: 50 µg) on the blot (Supplementary Figure 6D). Moreover, the detected size for the A_{2B}AR appeared to be larger (~46 kDa) compared to the A_{2B}AR detected in CHO-A_{2A}-A_{2B} (~37 kDa) or Jurkat-T cells (~37 kDa). Different glycosylation patterns may account for these differences. In contrast, the membrane preparations of recombinant HeLa cells transfected with the human A_{2A}AR (HeLa-A_{2A}) showed a higher expression level of A_{2A}ARs compared to that of A_{2B} ARs (Supplementary Figure 6D).

REFERENCES

1. Hu CD, Chinenov Y, Kerppola TK. Visualization of interactions among bZIP and Rel family proteins in living cells using bimolecular fluorescence complementation. Mol Cell. 2002; 9:789–98. https://doi.org/10.1016/S1097-2765(02)00496-3.
2. Simpson RJ. Homogenization of mammalian tissue. Cold Spring Harb Protoc. 2010; 2010:pdb.prot5455. https://doi.org/10.1101/pdb.prot5455.
3. Lodish HF, Berk A, Zipursky SL, Masudaira P, Baltimore D, Dranell J. Molecular Cell Biology. 4th edition. New York: W. H. Freeman; 2000. Section 5.2, Purification of Cells and Their Parts. Available from: https://www.ncbi.nlm.nih.gov/books/NBK21492/.
4. Bradford MM. A rapid and sensitive method for the quantitation of microgram quantities of protein utilizing the principle of protein-dye binding. Anal Biochem. 1976; 72:248–54. https://doi.org/10.1016/0003-2697(76)90527-3.

5. Kerppola TK. Design and implementation of bimolecular fluorescence complementation (BiFC) assays for the visualization of protein interactions in living cells. Nat Protoc. 2006; 1:1278–86. https://doi.org/10.1038/nprot.2006.201.
6. Greenwood C, Ruff D, Kirvell S, Johnson G, Dhillon HS, Bustin SA. Proximity assays for sensitive quantification of proteins. Biomol Detect Quantif. 2015; 4:10–16. https://doi.org/10.1016/j.bdq.2015.04.002.
7. Bannur SV, Kulgod SV, Metkar SS, Mahajan SK, Sainis JK. Protein determination by ponceau S using digital color image analysis of protein spots on nitrocellulose membranes. Anal Biochem. 1999; 267:382–89. https://doi.org/10.1006/abio.1998.3020.
8. Klotz KN, Hessling J, Hegler J, Owman C, Kull B, Fredholm BB, Lohse MJ. Comparative pharmacology of human adenosine receptor subtypes - characterization of stably transfected receptors in CHO cells. Naunyn Schmiedebergs Arch Pharmacol. 1998; 357:1–9. https://doi.org/10.1007/PL00005131.
9. Schiedel AC, Lacher SK, Linnemann C, Knolle PA, Müller CE. Antiproliferative effects of selective adenosine receptor agonists and antagonists on human lymphocytes: evidence for receptor-independent mechanisms. Purinergic Signal. 2013; 9:351–65. https://doi.org/10.1007/s11302-013-9354-7.
10. Pettengill MA, Lam VW, Ojcius DM. The danger signal adenosine induces persistence of chlamydial infection through stimulation of A2b receptors. PLoS One. 2009; 4:e8299. https://doi.org/10.1371/journal.pone.0008299.
11. De Filippo E, Namasivayam V, Zappe L, El-Tayeb A, Schiedel AC, Müller CE. Role of extracellular cysteine residues in the adenosine A2A receptor. Purinergic Signal. 2016; 12:313–29. https://doi.org/10.1007/s11302-016-9506-7.
12. Borrmann T, Hinz S, Bertarelli DC, Li W, Florin NC, Scheiff AB, Müller CE. 1-alkyl-8-(piperazine-1-sulfonyl) phenylxanthines: development and characterization of adenosine A2B receptor antagonists and a new radioligand with subnanomolar affinity and subtype specificity. J Med Chem. 2009; 52:3994–4006. https://doi.org/10.1021/jm900413e.
13. Seibt BF, Schiedel AC, Thimm D, Hinz S, Sherbiny FF, Müller CE. The second extracellular loop of GPCRs determines subtype-selectivity and controls efficacy as evidenced by loop exchange study at A2 adenosine receptors. Biochem Pharmacol. 2013; 85:1317–29. https://doi.org/10.1016/j.bcp.2013.03.005.
14. Schiedel AC, Hinz S, Thimm D, Sherbiny F, Borrmann T, Maass A, Müller CE. The four cysteine residues in the second extracellular loop of the human adenosine A2B receptor: role in ligand binding and receptor function. Biochem Pharmacol. 2011; 82:389–99. https://doi.org/10.1016/j.bcp.2011.05.008.
15. Hinz S, Lacher SK, Seibt BF, Müller CE. BAY60-6583 acts as a partial agonist at adenosine A2B receptors. J Pharmacol Exp Ther. 2014; 349:427–36. https://doi.org/10.1124/jpet.113.210849.
16. Fredholm BB, IJzerman AP, Jacobson KA, Klotz KN, Linden J. International Union of Pharmacology. XXV. Nomenclature and classification of adenosine receptors. Pharmacol Rev. 2001; 53:527–52.
17. Gao ZG, Balasubramanian R, Kiselev E, Wei Q, Jacobson KA. Probing biased/partial agonism at the G protein-coupled A(2B) adenosine receptor. Biochem Pharmacol. 2014; 90:297–306. https://doi.org/10.1016/j.bcp.2014.05.008.
18. Cooper J, Hill SJ, Alexander SP. An endogenous A2B adenosine receptor coupled to cyclic AMP generation in human embryonic kidney (HEK 293) cells. Br J Pharmacol. 1997; 122:546–50. https://doi.org/10.1038/sj.bjp.0701401.
19. Linden J, Thai T, Figler H, Jin X, Robeva AS. Characterization of human A(2B) adenosine receptors: radioligand binding, western blotting, and coupling to G(q) in human embryonic kidney 293 cells and HMC-1 mast cells. Mol Pharmacol. 1999; 56:705–13.
20. Phelps PT, Anthes JC, Correll CC. Characterization of adenosine receptors in the human bladder carcinoma T24 cell line. Eur J Pharmacol. 2006; 536:28–37. https://doi.org/10.1016/j.ejphar.2006.02.046.
21. Feoktistov I, Goldstein AE, Ryzhov S, Zeng D, Belardinelli L, Voyno-Yasenetskaya T, Biaggioni I. Differential expression of adenosine receptors in human endothelial cells: role of A2B receptors in angiogenic factor regulation. Circ Res. 2002; 90:531–38. https://doi.org/10.1161/01.RES.0000012203.21416.14.
22. van der Ploeg I, Ahlberg S, Parkinson FE, Olsson RA, Fredholm BB. Functional characterization of adenosine A2 receptors in Jurkat cells and PC12 cells using adenosine receptor agonists. Naunyn Schmiedebergs Arch Pharmacol. 1996; 353:250–60. https://doi.org/10.1007/BF00168626.
23. Wei Q, Costanzi S, Balasubramanian R, Gao ZG, Jacobson KA. A2B adenosine receptor blockade inhibits growth of prostate cancer cells. Purinergic Signal. 2013; 9:271–80. https://doi.org/10.1007/s11302-012-9350-3.
24. Hajiahmadi S, Panjehpour M, Aghaei M, Mousavi S. Molecular expression of adenosine receptors in OVCAR-3, Caov-4 and SKOV-3 human ovarian cancer cell lines. Res Pharm Sci. 2015; 10:43–51.
25. Panjehpour M, Castro M, Klotz KN. Human breast cancer cell line MDA-MB-231 expresses endogenous A2B adenosine receptors mediating a Ca2+ signal. Br J Pharmacol. 2005; 145:211–18. https://doi.org/10.1038/sj.bjp.0706180.
26. Hide I, Padgett WL, Jacobson KA, Daly JW. A2A adenosine receptors from rat striatum and rat pheochromocytoma PC12 cells: characterization with radioligand binding and by activation of adenylate cyclase. Mol Pharmacol. 1992; 41:352–59.

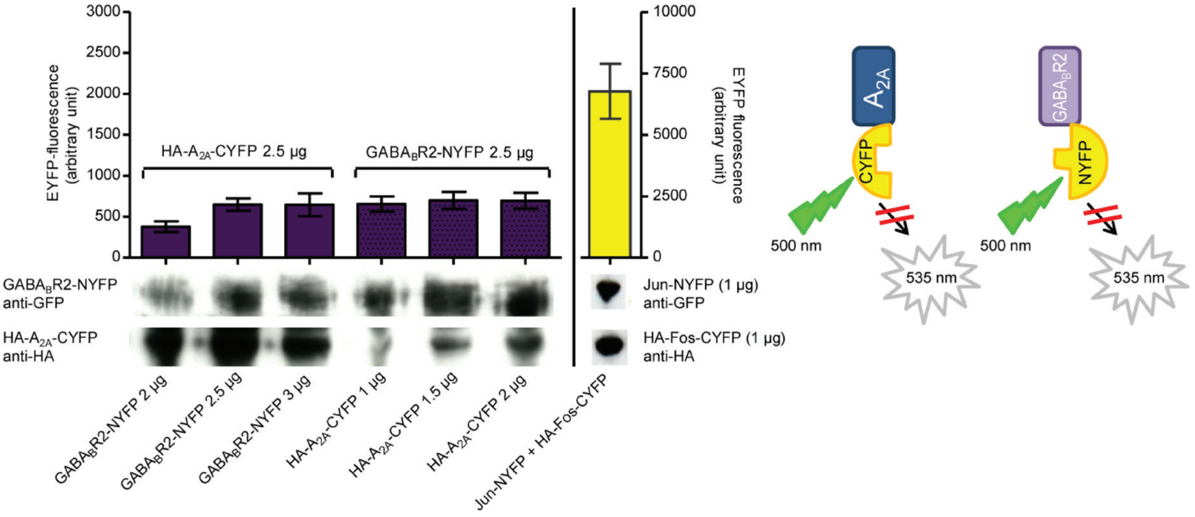

Supplementary Figure 1: BiFC assay and detection of protein expression with Western Blot including negative and positive controls. CHO-K1 cells were transiently co-transfected with a constant amount of plasmid DNA of HA-A_{2A}-CYFP (2.5 µg) and increasing amounts of $GABA_{B2}$-NYFP (2–3 µg), or a constant amount of plasmid DNA of $GABA_{B2}$-NYFP (2.5 µg) and increasing amounts of HA-A_{2A}-CYFP (1–2 µg) (negative controls). CHO-K1 cells were transiently co-transfected with 1 µg of Jun-NYFP and 1 µg of HA-Fos-CYFP to function as positive control. EYFP fluorescence was measured following excitation of the cells (20 µg of protein in 100 µl) at 500 nm. Non-transfected CHO-K1 cells (20 µg/100 µl) were used for determination of the background fluorescence, and the signal was substracted from that obtained for transfected cells. Data are shown as means ± SEM of 4–5 independent experiments performed in duplicates. The expression of both receptor constructs was confirmed by immunoblotting (25 µg/25 µl) using different primary antibodies (anti-GFP for $GABA_{B2}$-NYFP receptor (~124 kDa) and anti-HA for HA-A_{2A}-CYFP receptor (~56 kDa). The expression of both transcription factors was confirmed by immunoblotting (10 µg/10 µl) using different primary antibodies (anti-GFP for Jun-NYFP (~32 kDa) and anti-HA for HA-Fos-CYFP (~24 kDa). All blots are representative of three independent experiments.

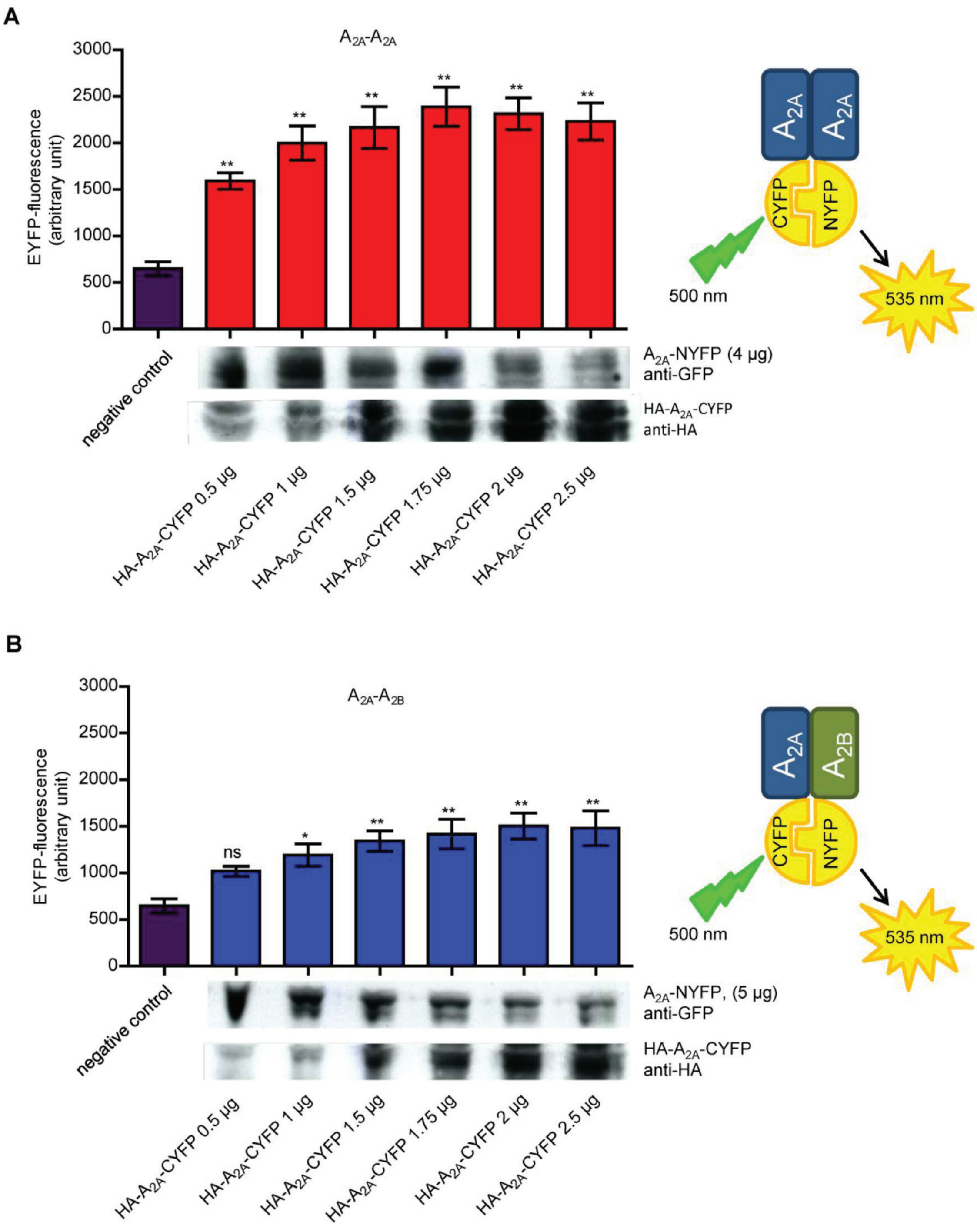

Supplementary Figure 2: BiFC assays to confirm A_{2A} and A_{2B}AR heteromerization. (**A**) CHO cells were transiently co-transfected with a constant amount of plasmid DNA for A_{2A}-NYFP (4 µg) and increasing amounts of cDNA for HA-A_{2A}-CYFP (0.5–2.5 µg). CHO cells transiently co-transfected with a constant amount of plasmid DNA for $GABA_{B2}$-NYFP (2.5 µg) and HA-A_{2A}-CYFP (2.5 µg) were used as a negative control. Data are means ± SEM of 5 independent experiments performed in duplicates. The one-way ANOVA with Dunnett's post-hoc test showed significant differences compared to the negative control. $^{**}p < 0.01$, $n = 5$. The expression of the receptors was confirmed by Western blotting (A_{2A}-NYFP receptor ~65 kDa, HA-A_{2A}-CYFP receptor ~56 kDa). Blots are representative of three independent experiments. (**B**) CHO cells were transiently co-transfected with a constant amount of plasmid DNA for A_{2B}-NYFP (5 µg) and increasing amounts of cDNA for HA-A_{2A}-CYFP (0.5–2.5 µg). CHO cells transiently co-transfected with constant amounts of plasmid DNA for $GABA_{B2}$-NYFP (2.5 µg) and HA-A_{2A}-CYFP (2.5 µg) were used as a negative control. Data are means ± SEM of 5 independent experiments performed in duplicates. The one-way ANOVA with Dunnett's post-hoc test showed significant differences compared to the negative control. ns: not significant, $^{*}p < 0.05$, $^{**}p < 0.01$, $n = 5$. The expression of both receptors was confirmed by Western blotting (A_{2B}-NYFP receptor ~56 kDa, HA-A_{2A}-CYFP receptor ~56 kDa). Blots are representative of three independent experiments.

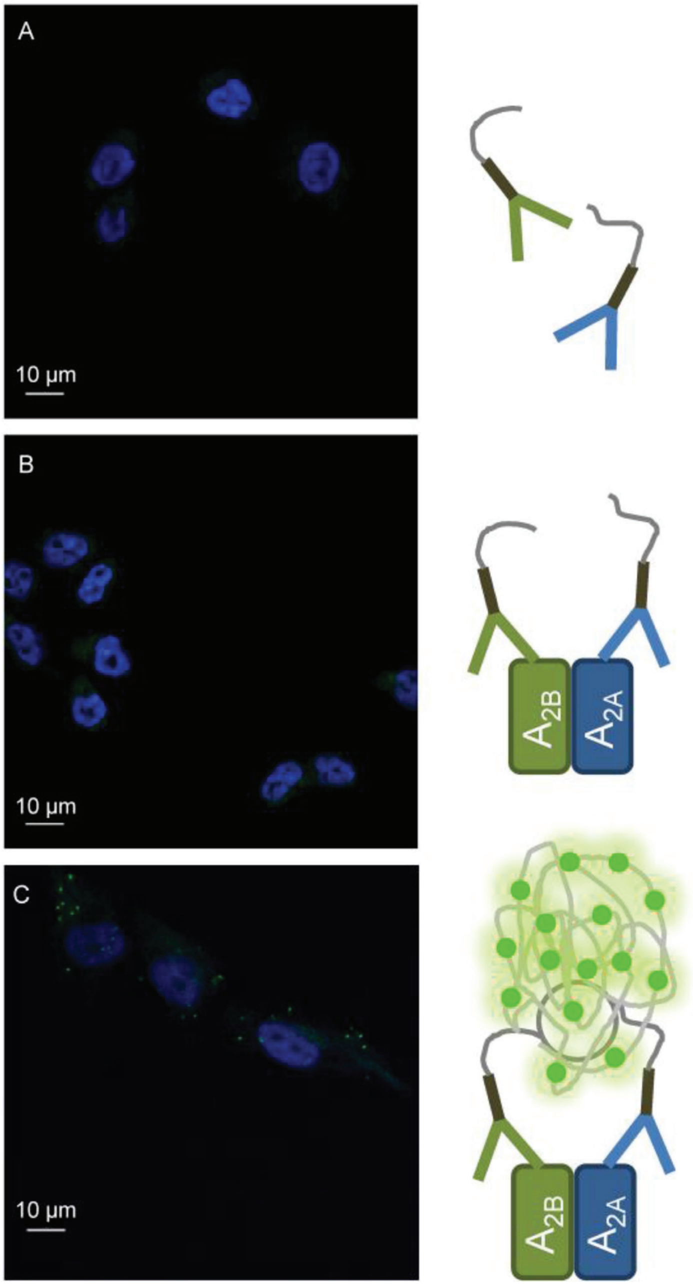

Supplementary Figure 3: *In situ* proximity ligation assay in a heterologous expression system. (**A**) Negative control using non-transfected CHO cells, which do not express human A_{2A} or A_{2B}ARs. No bright green fluorescent spots were visible; the results further show that the modified A_{2A} and A_{2B}AR primary antibodies do not bind to other proteins in an unspecific manner. (**B**) Negative control using CHO-A_{2A}-A_{2B} cells and omitting the ligation reaction. Accordingly, the sample cannot yield any bright green fluorescent spots and the low green signal is considered as background fluorescence. This sample was used as a reference for adjusting the lasers and filters to acquire the fluorescence images shown in (**C**). (**C**) Proximity ligation experiment using CHO-A_{2A}-A_{2B} cells. Stably transfected CHO-A_{2A}-A_{2B} cells were used in an *in situ* proximity ligation experiment. The green bright fluorescent spots are approximately 0.5 µm in diameter and represent a single heteromeric complex consisting of the human A_{2A} and A_{2B}AR.

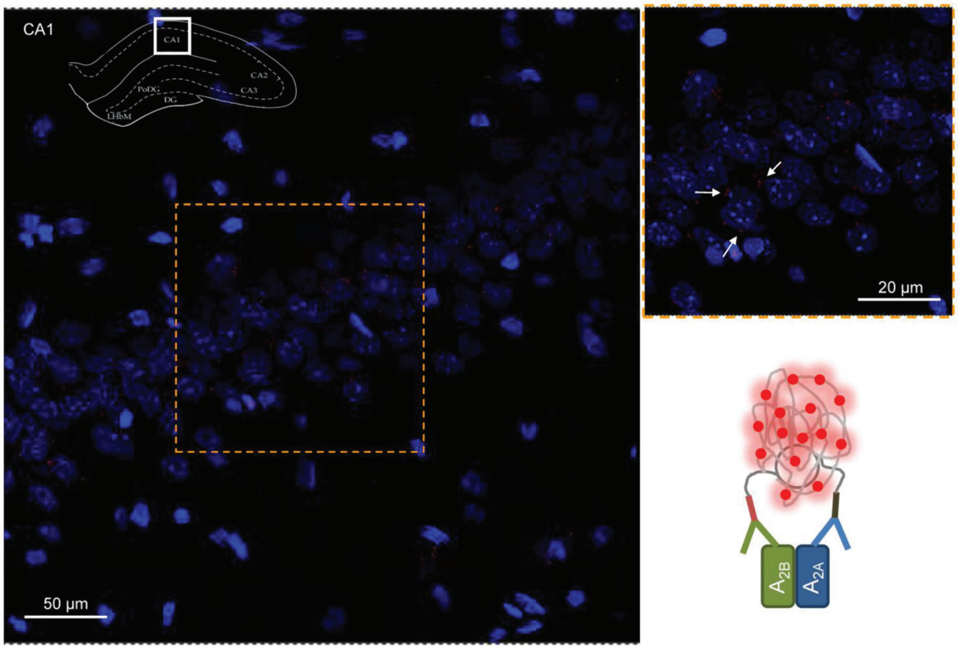

Supplementary Figure 4: A_{2A}-A_{2B} specific PLA clusters in the *CA1* region of the *dorsal hippocampus* of the rat (Bregma: −3.6 mm). The sampled region is taken from the rectangle of the dorsal *hippocampus* in the upper left part of the figure. The microphotographs taken are based on 20 Z-scans. The nuclei are shown in blue. A high density of PLA specific clusters in red are visualized mainly in the pyramidal cell layer shown in higher amplification in the panel of the upper right part. The arrows indicate their predominant perisomatic location. The diameter of the PLA clusters is mainly in the order of 0.5 to 1 µm. A low density of specific PLA clusters is also found in the radiatum and oriens close to the pyramidal cell layer.

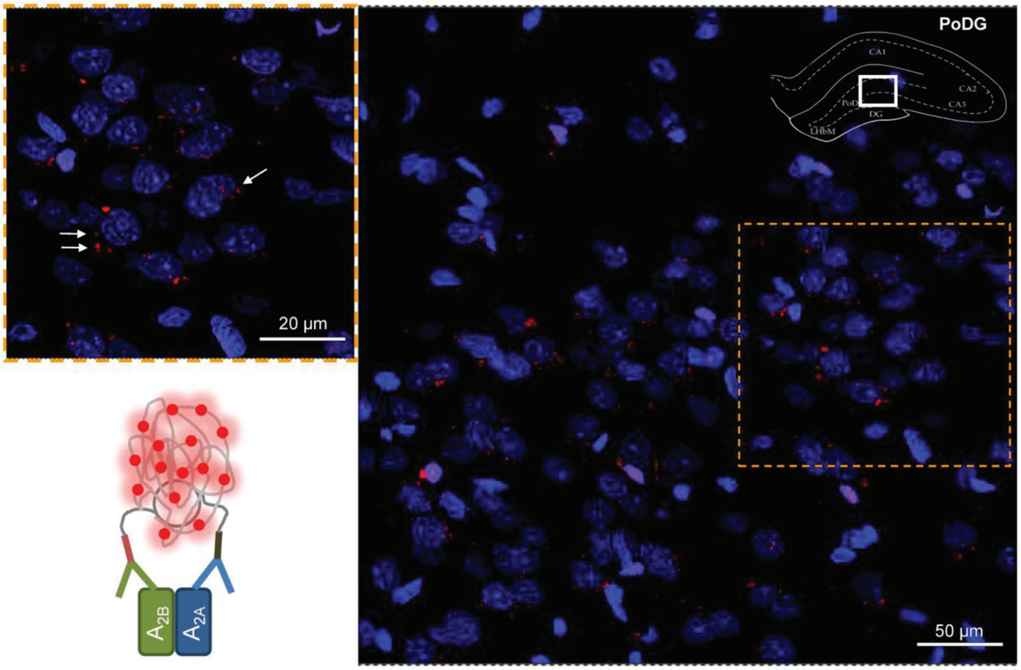

Supplementary Figure 5: A_{2A}-A_{2B}AR-specific PLA clusters in the polymorphic cell layer of the *dentate gyrus* (PoDG) of the rat dorsal *hippocampus* (Bregma: -3.6 mm). The sampled region is taken from the framed section of the dorsal hippocampus in the upper left corner of the figure. The microphotographs taken are based on 20 Z-scans. The nuclei are shown in blue. A high density of PLA-specific clusters in red is visualized in the polymorphic cell layer also shown in higher magnification in the upper left part of the figure. They are located mainly in a perisomatic position but also in the neuropil, and indicated by arrows. The diameter of the PLA clusters is mainly in the order of 0.5 to 2 µm.

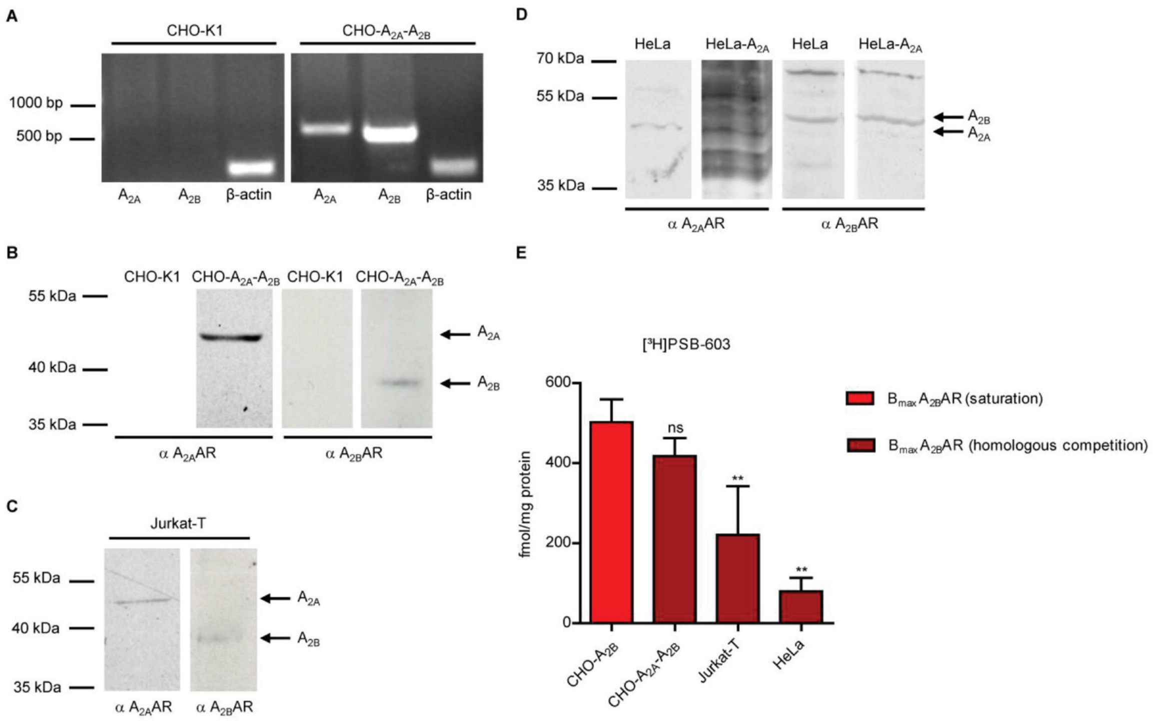

Supplementary Figure 6: Characterization of native and recombinant cell lines by reverse transcriptase (RT) PCR, Western blot analysis and radioligand binding studies. (**A**) mRNA analysis of non-transfected CHO cells and of CHO-A_{2A}-A_{2B} cells. Beta-actin: 258 bp, human A_{2A}AR: 491 bp, and human A_{2B}AR: 458 bp. In the CHO-K1 cDNA pool, only beta-actin was detected. The cDNAs of both AR subtypes and of beta-actin were detected in the CHO-A_{2A}-A_{2B} cell line ($A_{2B} > A_{2A}$). (**B**) Western blot analysis of non-transfected and co-transfected CHO cells. Both A_{2A}AR (~45 kDA, 25 µg membrane sample) and A_{2B}AR (~37 kDA, 10 µg membrane sample) were detectable in CHO-A_{2A}-A_{2B} cells (~$A_{2B} \geq A_{2A}$). (**C**) Western blot analysis of Jurkat-T cells. Both A_{2A}AR (~45 kDA, 50 µg membrane sample) and A_{2B}AR (~37 kDA, 10 µg membrane sample) were detectable in Jurkat-T cells (~$A_{2B} \geq A_{2A}$). (**D**) Western blot analysis of HeLa and HeLa-A_{2A} cells. Both A_{2A}ARs (~45 kDA, 50 µg membrane sample) and A_{2B}ARs (~46 kDA, 50 µg membrane sample) were detectable in HeLa cells (~$A_{2B} \geq A_{2A}$) and in HeLa-A_{2A} cells ($A_{2A} > A_{2B}$). All blots are representative of three independent experiments. (**E**) B_{max} values determined in saturation or in homologous competition binding experiments with the A_{2B}AR antagonist radioligand [³H] PSB-603. The calculated B_{max} values were 502 ± 57 fmol/mg protein for CHO-A_{2B} cells, (*60*), 418 ± 45 fmol/mg protein for CHO-A_{2A}-A_{2B} cells, 220 ± 122 fmol/mg protein for Jurkat-T cells, and 80 ± 34 fmol/mg protein for HeLa cells The one-way ANOVA with Dunnett's post-hoc test showed significant differences. ns: not significant, $^{**}p < 0.01$, $n = 2$–5.

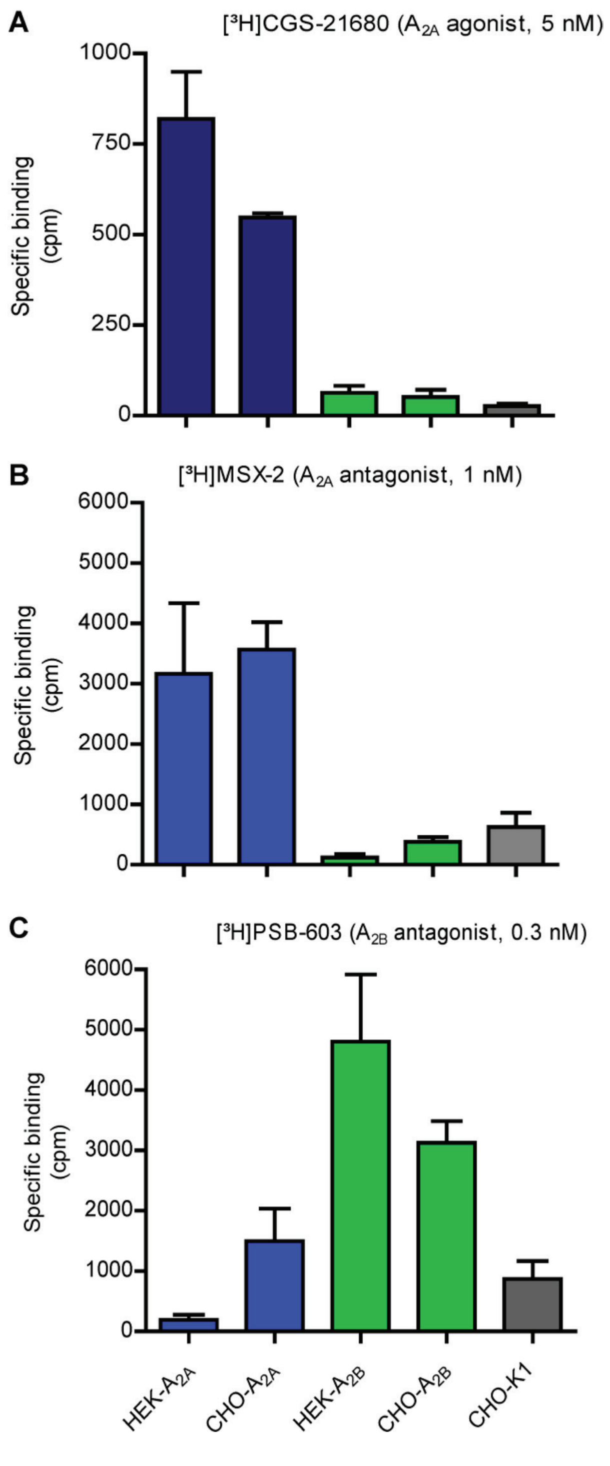

Supplementary Figure 7: (**A**) Specific binding in cpm of the A_{2A}-selective agonist radioligand [³H]CGS-21680 (5 nM), (**B**) the A_{2A}-selective antagonist radioligand [³H]MSX-2 (1 nM), and (**C**) the A_{2B}-selective antagonist radioligand [³H]PSB-603 (0.3 nM) using HEK-A_{2A} cell membranes (*n* = 2, in duplicates, 10 µg of protein per vial), CHO-A_{2A} cell membranes (*n* = 3, in duplicates, 100 µg of protein per vial), HEK-A_{2B} cell membranes (*n* = 2, in duplicates, 20 µg of protein per vial), CHO-A_{2B} cell membranes (*n* = 3, in duplicates, 100 µg of protein per vial), and CHO-K1 cell membranes (*n* = 3, in duplicates, 100 µg of protein per vial).

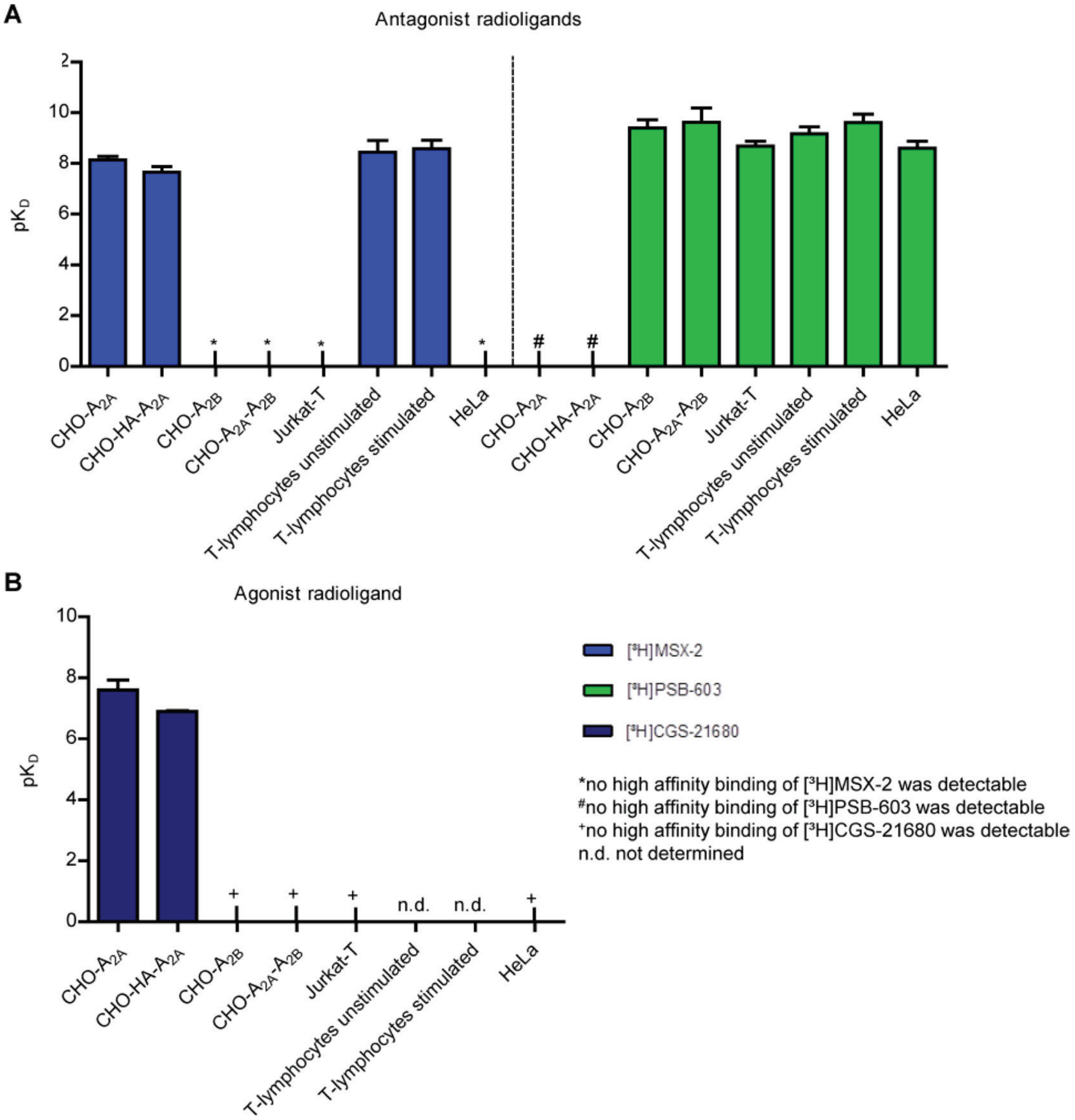

Supplementary Figure 8: K_D values for radiolabeled ligands in different cells expressing A_{2A} and A_{2B}ARs. Affinities for the selective antagonist (**A**) and agonist (**B**) radioligands at different cell lines. CGS-21680 and MSX-2 are A_{2A}-selective, while PSB-603 is A_{2B}-selective. K_D values were determined in saturation or homologous competition experiments, respectively (*n* = 2–4, see also Supplementary Table 2).

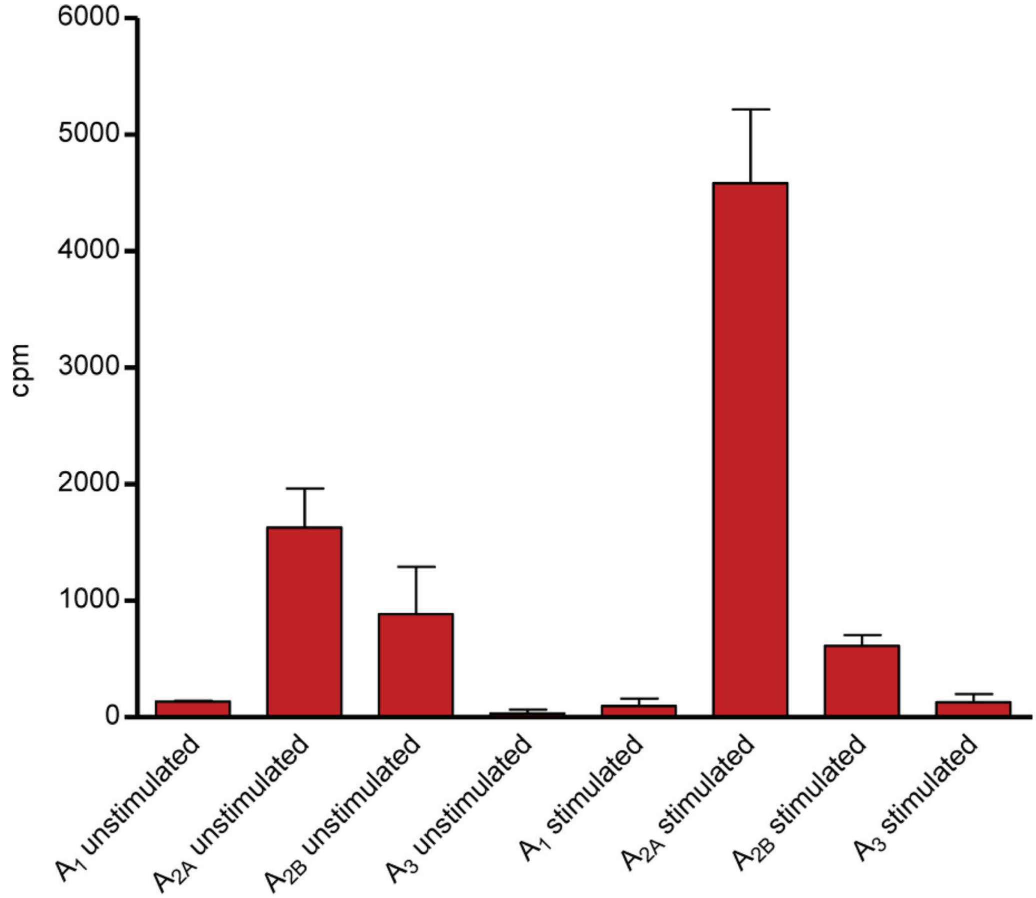

Supplementary Figure 9: Specific binding in cpm of the A_1 -selective antagonist radioligand [^{3}H]DPCPX (5 nM), the A_{2A}-selective antagonist radioligand [^{3}H]MSX-2 (5 nM), the A_{2B}-selective antagonist radioligand [^{3}H]PSB-603 (0.2 nM), and the A_3 -selective antagonist radioligand [^{3}H]PSB-11 (1 nM) at intact primary human lymphocytes that were either unstimulated or stimulated with phytohemeagglutinin (*n* = 3).

Supplementary Table 1: Relative mRNA and/or protein expression of human A_{2A}AR and human A_{2B}AR in the different cell lines

**Cell line**	**Relative expression profiles**			
	**A_{2A}AR mRNA**	**A_{2A}AR protein**	**A_{2B}AR mRNA**	**A_{2B}AR protein**
CHO	-[a]	-[b] [8]	-[a]	-[b] [8]
CHO-HA-A_{2A} ***(A_{2A})***	n. d.	+++[c]	n. d.	-
CHO-A_{2B} ***(A_{2B})***	n. d.	-	n. d.	++[c]
CHO-A_{2A}-A_{2B} ***($A_{2B} \geq A_{2A}$)***	++[a]	++[b]	+++[a]	++[b, c]
Jurkat-T ***($A_{2B} \geq A_{2A}$)***	++[a] [9]	+[b]	++[a] [9]	++[b, c]
HeLa ***($A_{2B} \geq A_{2A}$)***	++[a] [10]	+[b]	+++[a] [10]	+[b, c]
HeLa-HA-A_{2A} ***($A_{2A} > A_{2B}$)***	n. d.	+++[b, c]	n. d.	+[b]
T-Lymphocytes unstimulated ***($A_{2A} > A_{2B}$)***	+++[a] [9]	++[c]	+[a] [9]	+[c]
T-Lymphocytes stimulated ***($A_{2A} > A_{2B}$)***	+++[a] [9]	+++[c]	+[a] [9]	+[c]

n. d. not determined, [a]RT-PCR, [b]Western blot, [c]B_{max}-values.

Supplementary Table 2: Affinities of radioligands at different cell lines. K_{D}- and B_{max}-values were determined in saturation or homologous competition experiments (*n* = 2–4)

**Cell line**	**K_D ± SEM (nM)**		
	**[³H]CGS-21680**	**[³H]MSX-2**	**[³H]PSB-603**
CHO	No binding[c]	No binding[c]	No binding[c]
HEK or CHO-A_{2A} (A_{2A})	**25.0** ± 11.4[a]	**7.29** ± 1.08[a] (B_{max}: **212** ± 24 fmol/mg protein)	No binding[c]
CHO-HA-A_{2A} (A_{2A})	**127** ± 3[b] [11] (B_{max}: **478** ± 70 [11] fmol/mg protein)	**22.0** ± 5.9[b]	No binding[c]
HEK or CHO-A_{2B} (A_{2B})	No binding[c]	No binding[c]	**0.403** ± 0.188[a] [12] (B_{max}: **502** ± 57 [12] fmol/mg protein)
CHO-A_{2A}-A_{2B} ($A_{2B} \geq A_{2A}$)	No binding[c]	No binding[c]	**0.238** ± 0.072[b] (B_{max}: **418** ± 45 fmol/mg protein)
Jurkat-T ($A_{2B} \geq A_{2A}$)	No binding[c]	No binding[c]	**2.09** ± 0.72[b] (B_{max}: **220** ± 122 fmol/mg protein)
HeLa ($A_{2B} \geq A_{2A}$)	No binding[c]	No binding[c]	**2.49** ± 1.39[b] (B_{max}: **80** ± 34 fmol/mg protein)
HeLa-HA-A_{2A} ($A_{2A} > A_{2B}$)	**109** ± 38[b]	**21.9** ± 8.7[b]	n. d.
T-Lymphocytes unstimulated ***($A_{2A} > A_{2B}$)***	n. d.	**3.57** ± 2.67[b] (B_{max}: **3581** ± 2280 binding sites per cell)	**0.680** ± 0.242[b] (B_{max}: **3240** ± 815 binding sites per cell)
T-Lymphocytes stimulated ***($A_{2A} > A_{2B}$)***	n. d.	**2.58** ± 1.15[b] (B_{max}: **13588** ± 9939 binding sites per cell)	**0.241** ± 0.108[b] (B_{max}: **3994** ± 1343 binding sites per cell)

n. d. not determined

[a]saturation, [b]homologous competition, [c]no high affinity binding detectable.

Supplementary Table 3: Affinities of selected A_{2A} and A_{2B}AR agonists and antagonists at different cell lines (*n* = 2–6)

K_i ± SEM **(nM)**							
	**Adenosine**	**NECA**	**CGS-21680**	**BAY60-6583**	**MSX-2**	**PSB-603**	**Caffeine**
A_{2B}AR expressing cell lines (HEK or CHO)							
Binding vs. [^{3}H]PSB-603 (0.3 nM)	**2,680** ± 150	**1,890** ± 240 [59]	**>10,000** [13]	**212** ± 20 [14]	**>1000**	**0.553** ± 0.103 [12]	**33,800** ± 1,200 [12]
A_{2A}AR expressing cell lines (HEK or CHO)							
Binding vs. [^{3}H]MSX-2 (1 nM)	**212** ± 46	**11.7** ± 3.7	**249** ± 53	n. d.	**2.03** ± 1.05 [13]	**>10,000** [12]	**5,640** ± 540
Binding vs. [^{3}H]CGS-21680 (5 nM)	**76.7** ± 0.5	**4.09** ± 0.16	**25.0** ± 11.4 (K_D)	n. d.	**5.00** ± 0.60	n. d.	**23,400** ± 12,200
Transfected CHO cells (CHO-A_{2A}-A_{2B}), $A_{2B} \geq A_{2A}$							
Binding vs. [^{3}H]PSB-603 (0.3 nM)	**10,200** ± 1,400	**5,400** ± 363	n. d.	**107** ± 17	n. d.	**0.172** ± 0.053	**6,220** ± 860
[^{3}H]MSX-2 and [^{3}H]CGS-21680: Radioligands did not display high affinity binding							
Jurkat-T cells, ($A_{2B} \geq A_{2A}$)							
Binding vs. [^{3}H]PSB-603 (0.3 nM)	**>10,000**	**>10,000**	n. d.	**2,960** ± 1,220	n .d.	**1.43** ± 0.58	**>10,000**
[^{3}H]MSX-2 and [^{3}H]CGS-21680: Radioligands did not display high affinity binding							
***HeLa cells**, ($A_{2B} \geq A_{2A}$)*							
Binding vs. [^{3}H]PSB-603 (0.3 nM)	**>1,000**	**>10,000**	n. d.	**212** ± 58	n .d.	**1.43** ± 0.73	**>10,000**
[^{3}H]MSX-2 and [^{3}H]CGS-21680: Radioligands did not display high affinity binding							
***HeLa-A_{2A} cells**, ($A_{2A} > A_{2B}$)*							
Binding vs. [^{3}H]MSX-2 (1 nM)	**1,150** ± 289	**3,790** ± 800	**2,150** ± 260	**>1,000**	**21.9** ± 8.3	**>1,000**	**>10,000**
Binding vs. [^{3}H]CGS-21680 (5 nM)	**855** ± 86	**134** ± 41	**109** ± 36	>**1,000**	**20.1** ± 7.2	**>1,000**	**>10,000**

n. d. not determined.

Supplementary Table 4: EC_{50}/K_B-values of selected A_{2A} and A_{2B}AR agonists and antagonists determined in cAMP accumulation assays at different cell lines (*n* = 2–6)

	EC_{50}/K_B-value ± SEM (nM)						
	**Adenosine**	**NECA**	**CGS-21680**	**BAY60-6583**	**MSX-2[a]**	**PSB-603[a]**	**Caffeine[a]**
Transfected CHO cells (CHO-A_{2B})							
cAMP	**12,500** ± 660	**294** ± 70	**>100,000** [13]	**165** ± 36 [13]	n. d.	**0.358** ± 0.125	**15,600** ± 4,800
Transfected CHO cells (CHO-A_{2A})							
cAMP	**174** ± 30	**10.5** ± 0.8 [28]	**16.6** ± 0.1 [28]	**>10,000**	**14.5** ± 3.6	n. d.	**45,700** ± 9,890
Transfected CHO cells (CHO-A_{2A}-A_{2B}), ($A_{2B} \geq A_{2A}$)							
cAMP	**13,100** ± 1450	**1,090** ± 29	**>10,000**	**193** ± 30	n. d.	**0.673** ± 0.079	**9,900** ± 1120
Jurkat-T cells ($A_{2B} \geq A_{2A}$)							
cAMP	**>100,000**	**3,810** ± 560	**>10,000**	**low signal up to 10 µM** [15]	n. d.	**0.871**± 0.250	**34,100** ± 4,580

n. d. not determined.
[a]Antagonist potencies were determined versus the non-selective agonist NECA.

Supplementary Table 5: Data on potencies of AR agonists and antagonists found in the literature for human[a] A_{2A}-, A_{2B}-, and A_{2A}-A_{2B}-expressing cells determined in cAMP assays. See Supplementary_Table_5
