# Supplementary material for: Adenosine A2A receptor ligand recognition and signaling is blocked by A2B receptors
Source: Oncotarget. 2018 Feb 6;9(17):13593–611. doi: 10.18632/oncotarget.24423 (PMC5862601; doi:10.18632/oncotarget.24423)
Supplement: Supplementary file 2 [file oncotarget-09-13593-s002.docx]

**Supplementary Table 5: Data on potencies of AR agonists and antagonists found in the literature for human^a^ A_2A_-, A_2B_-, and A_2A_-A_2B_-expressing cells determined in cAMP assays.**

| **Cell line/ tissue (co)-expressing A_2A_ and A_2B_ receptors** | **Adenosine**  **EC_50_** | **NECA**  **EC_50_** | **CGS21680**  **EC_50_** | **BAY60-6583**  **EC_50_** | **A_2A_ antagonist**  **IC_50_/K_B_** | **A_2B_ antagonist**  **IC_50_/K_B_** | **Reference** |
| --- | --- | --- | --- | --- | --- | --- | --- |
| **Recombinant cells with single receptor expression** | | | | | | | |
| **CHO-A_2A_** | 730 nM | n. d. | n. d. | n. d. | n. d. | n. d. | Fredholm BB, et al. Biochem Pharmacol. 2001; 61: 443-448. [16] |
| **CHO-A_2A_** | 170 nM | 10.5 nM | 16.6 nM | n. d. | n. d. | n. d. | De Filippo E, et al. Purinergic Signal. 2016; 12: 313-329. [11] |
| **CHO-A_2B_** | 23,500 nM | n. d. | n. d. | n. d. | n. d. | n. d. | Fredholm BB, et al. Biochem Pharmacol. 2001; 61: 443-448. [16] |
| **CHO-A_2B_** | 11,900 nM | 109 nM | 31,100 nM | n. d. | n. d. | n. d. | De Filippo E, et al. Purinergic Signal. 2016;12: 313-329. [11] |
| **A_2B_ ≥ A_2A_** | | | | | | | |
| **HEK293**  **(native)** | n. d. | 331 nM | >10,000 nM | 296 nM | n. d. | n. d. | Gao Z, et al.  Biochem Pharmacol. 2014; 90: 297-306. [17] |
| **HEK293**  **(native)** | 65,000 nM | 5,750 nM | >>30,000 nM | n. d. | n. d. | DPCPX:  97.7 nM | Cooper J, et al.  Br J Pharmacol*.* 1997;  122: 546-550. [18] |
| **HEK-A_2B_** | n. d. | 21.0 nM | 2920 nM | 6.14 nM | n. d. | n. d. | Gao Z, et al.  Biochem Pharmacol. 2014; 90: 297-306. [17] |
| **HEK-A_2B_** | 2,510 nM | 200 nM | n. d. | 505 nM | n. d. | n. d. | Hinz S, et al.  J Pharmacol Exp Ther. 2014; 3: 427-436. [15] |
| **HEK-A_2B_** | n. d. | 28.6 nM | 15,200 nM | n. d. | n. d. | n. d. | Linden J, et al.  [Mol Pharmacol](https://www.ncbi.nlm.nih.gov/pubmed/10496952). 1999; 56: 705-713. [19] |
| **T24** human bladder carcinoma | 4,486 nM | 865 nM | >10,000 nM | n. d. | SCH58261:405 nM | MRS1754:  665 nM | Phelps PT, et al. Eur J Pharmacol. 2006; 536: 28-37. [20] |
| **T24** human bladder carcinoma | 4,620 nM | 242 nM | >10,000 nM | 43.0 nM | n. d. | n. d. | Gao Z, et al.  Biochem Pharmacol. 2014; 90: 297-306. [17] |
| **HMEC-1**  human microvascular endothelial cell line | n. d. | 14,000 nM | >100,000 nM | n. d. | n. d. | n. d. | Feoktistov I, et al. Circ Res. 2002; 90:531-538. [21] |
| **Jurkat-T** | n. d. | 5,800  nM | >100,000 nM | n. d. | n. d. | n. d. | Van der Ploeg et al.  Naunyn Schmiedeberg’s Arch Pharmacol*.*  1996; 353: 250-260. [22] |
| **Human prostatic carcinoma cells PC-3** | n. d. | 469 nM | >10,000 nM | 7.20 nM | n. d. | PSB-603: K_B_ =1 nM  versus BAY60-6583 | Wei Q, et al. [Purinergic Signal](https://www.ncbi.nlm.nih.gov/pmc/articles/PMC3646116/). 2013; 9: 271-280. [23] |
| **Ovarian cancer cell line**  **OVCAR-3** | n. d. | 6,600 nM | >>100,000 nM | n. d. | n. d. | n. d. | Hajiahmadi S, et al. [Res Pharm Sci](https://www.ncbi.nlm.nih.gov/pmc/articles/PMC4578211/). 2015; 10: 43-51. [24] |
| **Ovarian cancer cell line**  **Caov-4** | n. d. | 9,120 nM | >100,000 nM | n. d. | n. d. | n. d. | Hajiahmadi S, et al. [Res Pharm Sci](https://www.ncbi.nlm.nih.gov/pmc/articles/PMC4578211/). 2015; 10: 43-51. [24] |
| **Ovarian cancer cell line**  **SKOV-3** | n. d. | 18,000 nM | >100,000 nM | n. d. | n. d. | n. d. | Hajiahmadi S, et al. [Res Pharm Sci](https://www.ncbi.nlm.nih.gov/pmc/articles/PMC4578211/). 2015; 10: 43-51. [24] |
| **Breast cancer cell line**  **MDA-MB-231** | n. d. | 1,130 nM | >10,000 nM | n. d. | n. d. | n. d. | Panjehpour M, et al. [Br J Pharmacol](https://www.ncbi.nlm.nih.gov/pmc/articles/PMC1576131/). 2005; 145: 211-218. [25] |
| **A_2A_ > A_2B_** | | | | | | | |
| **HUVEC**  Human Umbilical Vein Endothelial Cells | n. d. | 950 nM | 469 nM | n. d. | n. d. | n. d. | Feoktistov I, et al. Circ Res. 2002; 90: 531-538. [21] |
| **PC12 (rat)**  **phaeochromocytoma** | n. d. | 200 nM | 90 nM | n. d. | n. d. | n. d. | van der Ploeg et al.  Naunyn Schmiedeberg’s Arch Pharmacol*.*  1996; 353: 250-260. [22] |
| **PC12 (rat)**  **phaeochromocytoma** | n. d. | Biphasic:  K_H_: 66 nM; K_L_: 7,500 nM | 64 nM | n. d. | n. d. | n. d. | Hide I, et al. Mol Pharmacol.  1992; 41: 352-359. [26] |

^a^In few cases, cells from other species are included as indicated.

n. d. not determined
